# Combined patch-clamp electrophysiology and single-cell genomic analysis reveal spiking tumor cells at the neocortical glioblastoma interface in humans

**DOI:** 10.1093/neuonc/noag059

**Published:** 2026-03-19

**Authors:** Tong Tong, Josephine D Hendriksen, Kirstine J Elbæk, Kata Molnar, Freja V Christiansen, Attila Ozsvar, Jens T Eschen, Francisco G Rodríguez-González, Ilayda D Pusat, Emilie Littau Christensen, Mads Rahbæk, Kathrine Pii Frederiksen, Søren O S Cortnum, Ann K Sindby, Kaare Meier, Nikola, Jens C H Sørensen, Jonathan T Ting, Marco Capogna, Bjarne W Kristensen, Jens R Nyengaard, Joachim Weischenfeldt, Wen-Hsien Hou, Anders R Korshøj

**Affiliations:** Department of Neurosurgery, Aarhus University Hospital, Aarhus, Denmark; Department of Clinical Medicine, Aarhus University, Aarhus, Denmark; Biotech Research and Innovation Center (BRIC), University of Copenhagen, Copenhagen, Denmark; Finsen Laboratoriet, Copenhagen University Hospital—Rigshospitalet, Copenhagen, Denmark; Department of Neurosurgery, Aarhus University Hospital, Aarhus, Denmark; Department of Clinical Medicine, Aarhus University, Aarhus, Denmark; Core Center for Molecular Morphology, Department of Clinical Medicine, Aarhus University, Aarhus, Denmark; Sino-Danish College (SDC), University of Chinese Academy of Sciences, Beijing, China; Core Center for Molecular Morphology, Department of Clinical Medicine, Aarhus University, Aarhus, Denmark; Department of Clinical Medicine, Aarhus University, Aarhus, Denmark; Department of Biomedicine, Aarhus University, Aarhus, Denmark; Department of Neurosurgery, Aarhus University Hospital, Aarhus, Denmark; Department of Clinical Medicine, Aarhus University, Aarhus, Denmark; Biotech Research and Innovation Center (BRIC), University of Copenhagen, Copenhagen, Denmark; Finsen Laboratoriet, Copenhagen University Hospital—Rigshospitalet, Copenhagen, Denmark; Department of Neurosurgery, Aarhus University Hospital, Aarhus, Denmark; Department of Clinical Medicine, Aarhus University, Aarhus, Denmark; Department of Neurosurgery, Aarhus University Hospital, Aarhus, Denmark; Department of Clinical Medicine, Aarhus University, Aarhus, Denmark; Department of Neurosurgery, Aarhus University Hospital, Aarhus, Denmark; Department of Clinical Medicine, Aarhus University, Aarhus, Denmark; Department of Neurosurgery, Aarhus University Hospital, Aarhus, Denmark; Department of Clinical Medicine, Aarhus University, Aarhus, Denmark; Department of Neurosurgery, Aarhus University Hospital, Aarhus, Denmark; Department of Neurosurgery, Aarhus University Hospital, Aarhus, Denmark; Department of Neurosurgery, Aarhus University Hospital, Aarhus, Denmark; Department of Clinical Medicine, Aarhus University, Aarhus, Denmark; Allen Institute for Brain Science, Seattle, WA, USA; Department of Biomedicine, Aarhus University, Aarhus, Denmark; Department of Pathology, The Bartholin Institute, Rigshospitalet, Copenhagen University Hospital, Copenhagen, Denmark (B.W.K.); Biotech Research and Innovation Center (BRIC), University of Copenhagen, Copenhagen, Denmark; DCCC Brain Tumor Center, Rigshospitalet, Copenhagen University Hospital, Copenhagen, Denmark; Core Center for Molecular Morphology, Department of Clinical Medicine, Aarhus University, Aarhus, Denmark; Department of Pathology, Aarhus University Hospital, Aarhus, Denmark; Biotech Research and Innovation Center (BRIC), University of Copenhagen, Copenhagen, Denmark; Finsen Laboratoriet, Copenhagen University Hospital—Rigshospitalet, Copenhagen, Denmark; DCCC Brain Tumor Center, Rigshospitalet, Copenhagen University Hospital, Copenhagen, Denmark; Department of Biomedicine, Aarhus University, Aarhus, Denmark; Department of Neurosurgery, Aarhus University Hospital, Aarhus, Denmark; Department of Clinical Medicine, Aarhus University, Aarhus, Denmark; DCCC Brain Tumor Center, Rigshospitalet, Copenhagen University Hospital, Copenhagen, Denmark

**Keywords:** action potential, cancer neuroscience, glioblastoma, organotypic slice culture, patch-seq

## Abstract

**Background:**

Electrophysiological features of glioblastoma cells (GBCs) remain largely elusive, challenging our comprehension of glioblastoma pathophysiology. Spiking GABAergic-oligodendrocyte-progenitor (OPC) tumor cells were recently described in IDH-mutant glioma, correlating with prolonged patient survival. Here, we characterize single-cell features at the neocortical leading edge (LE) of glioblastoma patients using combined electrophysiological, morphological, and transcriptomic profiling.

**Methods:**

We examined GBCs and non-tumor cells using acute and cultured organotypic slices of cancer-infiltrated neocortical tissues from glioblastoma patients. Electrophysiological properties of LE cells were investigated using whole-cell patch-clamp recording, with dye loading to characterize single-cell morphology. We used Patch-seq to determine the transcriptomic features of recorded LE cells and discriminate tumor and non-tumor cells, followed by gene set enrichment analysis and CellChat to identify differential gene expression and signaling.

**Results:**

Upon depolarization, more than half of LE cells show aberrant action potentials (aAPs), akin to neurodevelopmental cells. Reconstructed LE cells have abnormal somatodendritic morphology. Patch-seq revealed that GBCs and non-tumor cells share a similar electrophysiological phenotype, including aAP generation, depolarized membrane potential, and elevated input resistance. Transcriptomic analysis shows that the aAP phenotype occurs across diverse GBC states and correlates with lower enrichment of proliferation-related pathways at the single-cell level, but higher enrichment of inflammatory/immune, angiogenic, and mesenchymal transition pathways. Non-tumor cells exhibit hybrid transcriptomic signatures, with predominantly neuronal but minor enrichment of astrocytic features.

**Conclusion:**

We find electrophysiological aAP behavior of GBCs in human glioblastoma, closely resembling that of hybrid cells in IDH-mutant glioma, supporting the hypothesis of neuronal mimicry across different glioma types.

## Graphical Abstract

**Graphical Abstract:**
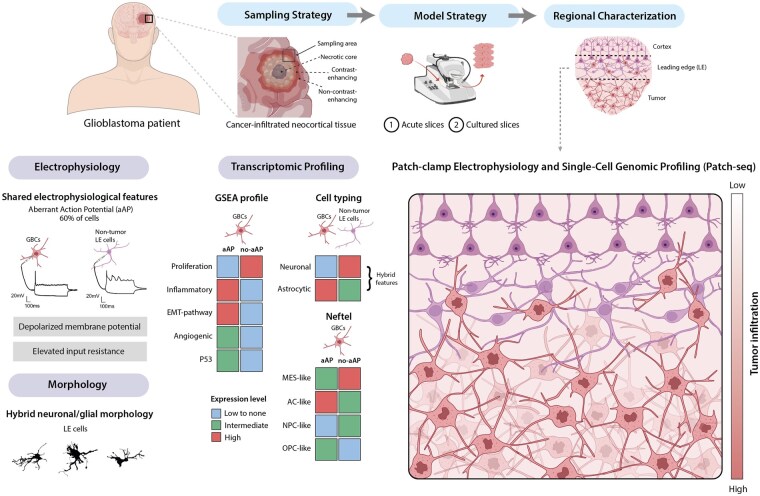

Key Points

- Cortical glioblastoma cells fire aberrant action potentials and share electrophysiological phenotypes with non-tumor cells in humans.
- Spiking glioblastoma cells are heterogeneous but exhibit reduced proliferation and increased inflammatory signaling.

Importance of the Study

This study sheds light on the diverse pathophysiological and molecular features of cells in the neocortical infiltration zone of glioblastoma. Using patient-derived brain/tumor slices in vitro, we unveiled the electrical properties of cells at the leading edge (LE). More than half of LE cells displayed aberrant action potentials (aAPs), and patch-seq analysis further showed that glioblastoma cells (GBCs) and non-tumor cells shared indistinguishable passive- and aAP properties, including resting potential, input resistance, initiation threshold, amplitude, rise- and decay kinetics, indicating a universal functional feature of the LE microenvironment. aAP GBCs showed higher cell–cell interactions, elevated inflammatory, angiogenic, and mesenchymal transition pathway signaling, and reduced proliferation signaling compared to GBCs without the aAP phenotype (no-aAP), suggesting a non-dividing cell state with potential involvement in diverse cancer mechanisms, including tumor-immune regulation and network integration. Our findings align with recent studies in IDH-mutant glioma and point to electrically active GBCs and cellular hyperexcitabil-ity features as important attributes of glioma pathophysiology.

Glioblastoma is the most common malignant primary brain tumor in adults.^1^^,^^2^ These tumors display significant heterogeneity^3–5^ and resistance to treatment, leaving patients facing a dismal prognosis. Recent discoveries in cancer neuroscience revealed that glioblastoma cells (GBCs) hijack neuronal networks and mechanisms to promote disease growth. GBC proliferation and brain invasion are facilitated by excitatory^6–8^ glutamatergic,^9^^,^^10^ cholinergic,^11–13^ and serotonergic^14^ neuron-to-glioma synapses and further augmented by paracrine neuroligin-3^15–17^ and brain-derived neurotrophic factor signaling.^18^ Moreover, pacemaker-like GBCs within the densely interconnected tumor network propagate intercellular Ca^2+^ waves, driving malignant self-repair and therapy resistance.^6–8^ Recent studies detailing the spatial molecular organization of glioblastoma further show that synaptic gene programs and neurodevelopmental malignant cell states, that is, neural-progenitor-like (NPC-like), oligodendrocyte-progenitor-like (OPC-like), and astrocyte-like (AC-like) GBCs, are increasingly enriched at the infiltrating leading edge (LE) of the tumor.^9^^,^^10^ Accordingly, unconnected NPC-/OPC-like cells in this region proliferate and colonize the surrounding brain, stimulated by synaptic input.^11–13^

Bridging the molecular and functional observations of tumor cells is a current imperative in cancer neuroscience. Despite the abovementioned intriguing features, the electrophysiological properties of GBCs remain largely elusive. Specifically, it is unclear if some GBCs have excitable membranes, potentially enabling active electrical integration into the surrounding tumor network and adjacent brain. While some studies have indicated that GBCs do not fire action potentials,^9^^,^^10^ a recent study by Curry et al. identified single, short action potential-generating tumor cells with hybrid NPC/glial morphological and electrophysiological features and GABAergic OPC transcriptomic profiles in human IDH-mutant gliomas, using deep single-cell profiling.^19^ Moreover, the presence of spiking glioma cells conferred increased patient survival, indicating a significant functional importance of this feature. Interestingly, the authors did not observe spiking behavior in IDH-wildtype gliomas, although the small sample size precluded definitive conclusions.

Here, we aimed to elucidate this further and characterize intrinsic electrophysiological and genetic profiles of diverse cells in the human glioblastoma-neocortical interface. To this end, we applied an approach analogous to Curry et al.,^19^ using acute slices and stable human organotypic slice cultures derived from glioblastoma patients. This platform enabled detailed characterization of the electrophysiological, morphological, and transcriptomic properties of both GBCs and non-tumor cells in the neocortical LE region (LE cells), as well as neurons in the adjacent non-infiltrated neocortex (non-LE neurons). Multimodal cellular signatures were generated using patch-seq, integrating whole-cell patch-clamp recordings with single-cell transcriptomic profiling. This method allowed direct coupling between electrophysiological phenotype and gene expression identity within the same cell, providing critical mechanistic insight into the cellular heterogeneity landscape and functional relevance of cells in the neocortical tumor microenvironment.

## Methods

### Human Glioblastoma Tissue Acquisition

**Figure 1.**
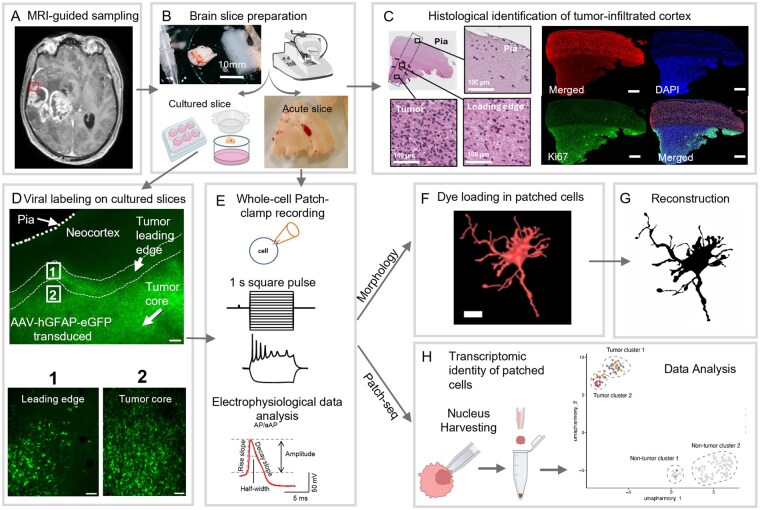
Workflow for utilizing patient-derived tumor-infiltrated neocortical tissue. (A) The box highlights the tumor-infiltrated neocortical region with contrast-enhanced region on T1 MRI for neurosurgical resection. Tissues from this region were used for downstream ex vivo experimentation. (B) Schematic overview of tissue processing. Surgically resected glioblastoma tumor-infiltrated cortical specimens were prepared for acute and organotypic slice cultures using a vibratome. Slices were either used as acute preparations for immediate patch-clamp recordings or maintained in culture for subsequent viral transduction. Created with BioRender.com (C) A representative image showing hematoxylin and eosin (H&E) staining of a tumor-infiltrated cortical brain slice from a glioblastoma patient. The increased cellular density indicates tumor infiltration. Immunohistochemistry showing the target marker with NeuN (neuronal marker), DAPI (nuclei marker), and Ki67 (proliferation marker) in tumor-infiltrated human neocortical slices. Co-staining confirms the presence of infiltrating tumor cells within the tissue (scale bar: 500 µm). (D) Cell-type-specific viral transduction using adeno-associated virus (AAV) vectors. Example demonstrating the use of the GFAP promoter to target astrocytes or glioblastoma cells (GFAP-eGFP⁺), allowing for fluorescence-based identification of labeled cells. (E) Whole-cell patch-clamp recordings were performed on either acute slices or fluorescence-guided cultured slices to characterize the intrinsic membrane properties of identified cell types. Electrophysiological data were compiled for joint analysis using established parameters of intrinsic membrane properties. (F) Seven patched cells were filled with Alexa Fluor 594 during recordings. Confocal *z*-stacks were acquired for 3D morphological reconstruction of the recorded cells, allowing detailed structural analysis. An example 3D reconstruction is shown. (G) An example traced cell morphology is shown. (H) Following successful electrophysiological recordings, the nucleus of the patched cell was aspirated into the pipette and processed for single-nucleus RNA sequencing. To enhance transcriptomic coverage, nuclei from neighboring unpatched cells were also collected. The resulting transcriptomic data were analyzed to correlate intrinsic excitability with gene expression profiles. Created with BioRender.com

Human tissue procedures were approved by the Central Denmark Region Committee for Health Research Ethics (Journal Number: 1-10-72-82-17) and adhered to the World Medical Association Declaration of Helsinki principles.^20^ With patient consent, cortical tumor-infiltrated tissue was resected from 18 glioblastoma patients at Aarhus University Hospital, with diagnoses confirmed by WHO 2021 criteria^2^ (Supplementary Figure S1). Samples were resected from cortical transition zones between contrast-enhancing and non-enhancing regions on T1-weighted MRI (Figure 1A), retaining intact pia mater and showing 5-ALA or sodium-fluorescein fluorescence during microsurgical resection to validate tumor infiltration.^21^^,^^22^ Tissue specimens (1-2 cm^3^) were immediately immersed in ice-cold artificial cerebrospinal fluid (ACSF) containing (mM): 75 sucrose, 84 NaCl, 2.5 KCl, 1 NaH_2_PO_4_, 25 NaHCO_3_, 25 d-glucose, 0.5 CaCl_2_·2H_2_O, and 4 MgCl_2_·6H_2_O, with a pH range of 7.3-7.4. The samples were transported to the laboratory in carbogenated ACSF (95% O_2_, 5% CO_2_) within 30 minutes.

### Acute and Cultured Human Organotypic Slice Preparation

The human ex vivo brain slice culture method was adapted from established methods.^23–25^ 350-μm-thick slices were cut in ice-cold carbogenated ACSF using a vibratome (Leica 1200S). After a 30-minute recovery in carbogenated ACSF at 34°C, slices were transferred to ACSF containing (mM): 130 NaCl, 3.5 KCl, 1 NaH_2_PO_4_, 24 NaHCO_3_, 10 d-glucose, 1 CaCl_2_·2H_2_O, and 3 MgCl_2_·6H_2_O with a pH range of 7.3-7.4, at room temperature. The slices were then divided either for acute recordings or cultured on membrane inserts in 6-well plates for virus transduction. The slice culture media contained (mM): 8.4 g/L MEM Eagle medium, 20% heat-inactivated horse serum, 30 HEPES, 13 d-glucose, 15 NaHCO_3_, 1 ascorbic acid, 2 MgSO_4_·7H_2_O, 1 CaCl_2_·4H_2_O, 0.5 GlutaMAX-I and 1 mg/L insulin, 25 U/mL penicillin/streptomycin, with a pH range of 7.2-7.3 adjusted by Tris-base, and osmolality of 295-305 mOsm/kg, and sterile-filtered. Medium was refreshed every 1-2 days until use.

### AAV-Viral Transduction

The adeno-associated virus (AAV) vectors were acquired from the University of Zurich Viral Vector Facility. AAV-hGFAP-EGFP (AAV5-hGFAP-hHEbl/E-EGFP-bGHp(A)) and AAV-hSyn1-EGFP (AAV1-hSyn-EGFP-WPRE-hGHp(A)) were used in this study. Viral titers ranged between 1 and 3 × 10^13^ units/mL. The viral vectors were applied to the slice surface using a fine micropipette at least 1 hour after the initial plating to allow the slice to equilibrate.

### Whole-Cell Patch-Clamp Recording

Tumor-infiltrated brain slices were placed in the recording chamber of a SliceScope microscope (Scientifica) and perfused with carbogenated ACSF at 32°C-35°C. A SciCam Pro camera (Scientifica) was used for visualization and image capture. Whole-cell recordings were performed using pulled borosilicate pipettes (5-7 MΩ; Harvard glass capillaries, GC120F-10) filled with an internal solution containing (mM): 126 K-gluconate, 10 HEPES, 4 Mg-ATP, 0.3 Na_2_-GTP, 4 KCl, 10 phosphocreatine, 8 biocytin, and 50 mM Alexa-594 with a pH range of 7.2-7.3 and 295 mOsm/kg osmolality. Data were acquired with a Multiclamp 700B and Digidata 1550B (Molecular Devices), low-pass filtered at 2 kHz, and digitized at 20 kHz. Cells were accepted if they had >1 GΩ seal resistance and access resistance <20 MΩ. Resting membrane potential (RMP) was measured after break-in, and APs were evoked by 3-ms depolarizing steps. Aberrant action potentials (aAPs) were defined by a derivative threshold (>20 mV/ms)^26^ and quantified for initiation, amplitude, half-width, and 10%-90% rise/decay slopes. To confirm ionic contributions, TEA (30 mM, Sigma-Aldrich) and TTX (1 µM, TOCRIS) were sequentially applied. Coordinates of recorded cells were saved for later histology in selected cases.

### Nucleus Collection Following Patch-Clamp Recordings

For a subset of recorded or neighboring cells, nuclei were extracted by applying gentle negative pressure at the end of recording, aspirating the nuclei and cytoplasm into the pipette, and subsequently expelling the material into lysis buffer with RNase inhibitor (Takara).^25^ Samples were snap-frozen on dry ice and stored at −80°C for transcriptomic processing.

### cDNA Amplification, Library Construction, and Sequencing

Following nucleus collection, reverse transcription and cDNA amplification were performed using the SMART-seq v2 Ultra Low Input RNA Kit (Takara). The cDNA quality and yield were assessed via Bioanalyzer (Agilent Technologies). Libraries were prepared using the Nextera XT DNA Library Preparation Kit (Illumina) and sequenced on Illumina NextSeq 500.

### RNA-Seq Gene Expression Quantification

Reads were aligned to GRCh38 using STAR v2.7.0c, with the parameters outFilterScoreMinOverLread = 0.3, outFilterMatchNminOverLread = 0.3, outFilterMatchNmin = 0, and outFilterMismatchNmax = 2 options. Gene counts were computed using the R Genomic Alignments summerizeOverlaps function with the “IntersectionNotEmpty” option.

### Transcriptomic Data Analysis

The count matrices were imported into R (version 4.1.0) and analyzed using Seurat (version 4.1.1).^27^ The cells underwent quality control and filtering, removing cells with more than 10% of transcripts mapping to mitochondrial genes or cells with less than 500 unique genes detected, resulting in a dataset of 144 cells. To distinguish between GBCs and non-tumor cells, CopyKat (version 1.1.0) and InferCNV (version 1.22.0, inferCNV of the Trinity CTAT Project. https://github.com/broadinstitute/inferCNV) were used to detect copy-number alterations (CNAs) in the cells. Consensus of cells showing CNA were annotated as GBCs, while the remaining cells underwent cell typing using label transfer with the glioblastoma Darmanis et al. dataset as reference.^28^ Integration across patients was performed using Harmony.^29^ For visualization, Uniform Manifold Approximation and Projection (UMAP) was calculated on the top 30 principal components. The GBCs were classified using the cellular states from Neftel et al.^30^ using AddModuleScore from Seurat, calculating the average expression level of each program subtracted by the average expression of a control gene set. Correspondingly, proliferation was scored in Seurat using module scores derived from the built-in S and G2/M phase gene sets.

### Differential Gene Expression Analysis

Differential gene expressions between aAP and no-aAP cells (both non-tumor cells and GBCs) were assessed using the MAST test implemented in Seurat’s FindMarkers function. Cells were subset to the tumor population, and the patient of origin was included as a latent variable to account for inter-sample variability. Gene set enrichment analysis (GSEA) was performed using the fgsea R package to compare pathways enriched between aAP and no-aAP GBCs. The ranked gene list from differential gene expression analysis was used as input. As a query, hallmark gene sets were obtained from the Molecular Signatures Database (MSigDB v2024.1). Cell–cell interaction analysis was performed using the CellChat R package version 2.1.2. The standard CellChat pipeline was used, with cells split into aAP and no-aAP groups, and communication probabilities calculated using triMean.

### DNA Extraction

See Supplementary Methods.

### Shallow Whole-Genome Sequencing Library Preparation and Copy Number Estimation

See Supplementary Methods.

### Correlation Between aAP Genetic Signatures Versus Survival

See Supplementary Methods.

### Hematoxylin and Eosin Staining for Tumor Area Identification

See Supplementary Methods.

### Immunohistochemistry

See Supplementary Methods.

### Antibodies and Morphology Reconstruction

See Supplementary Methods.

### Statistical Analysis

Statistical analyses were performed in R (version 4.1.0) or GraphPad Prism 6 (GraphPad Software). Data are presented as mean ± SEM unless otherwise indicated. Comparisons between 2 groups were conducted using unpaired 2-tailed Student’s *t*-tests. For comparisons involving more than 2 groups, 1-way ANOVA was used, followed by Tukey’s post hoc multiple comparisons test. When evaluating repeated or paired measures across 3 groups, a 2-way ANOVA followed by Tukey’s multiple comparison test was applied. Correlations of electrophysiological parameters were assessed with linear regression and Pearson’s correlation. The classification of APs and aAPs was established by assessing the distribution of individual electrophysiological parameters through a normality test, followed by hierarchical cluster analysis. The identification of significant changes in gene expression was performed in R using MAST.^31^ Levels of statistical significance are indicated as follows: *P < .05, **P < .01, ***P < .001, and ****P < .0001.

## Results

We investigated tumor-infiltrated samples of neocortical brain tissue obtained from glioblastoma patients during fluorescence-guided neurosurgical resection (Figure 1A; Supplementary Figure S1). The experimental workflow involved the preparation of acute brain slices and AAV-mediated cell labeling in cultured slices (Figure 1B). To identify regions of tumor infiltration, hematoxylin and eosin (H&E) and immunohistochemistry staining were performed on adjacent slices in a subset of patients (Figure 1C; Supplementary Figure S2). Whole-cell patch-clamp recordings were performed to obtain electrophysiological and morphological readouts from cells located at the tumor LE or non-tumor-infiltrated cortex in both acute and cultured slices (Figure 1D-G). A subset of recorded cells was loaded with an intracellular dye to enable morphological reconstruction (Figure 1F and G). In another subset, we performed patch-seq by harvesting the cytoplasm and nuclei for single-nucleus RNA sequencing and analysis (Figure 1H).

### Neocortical LE Cells Display Aberrant Action Potentials

**Figure 2.**
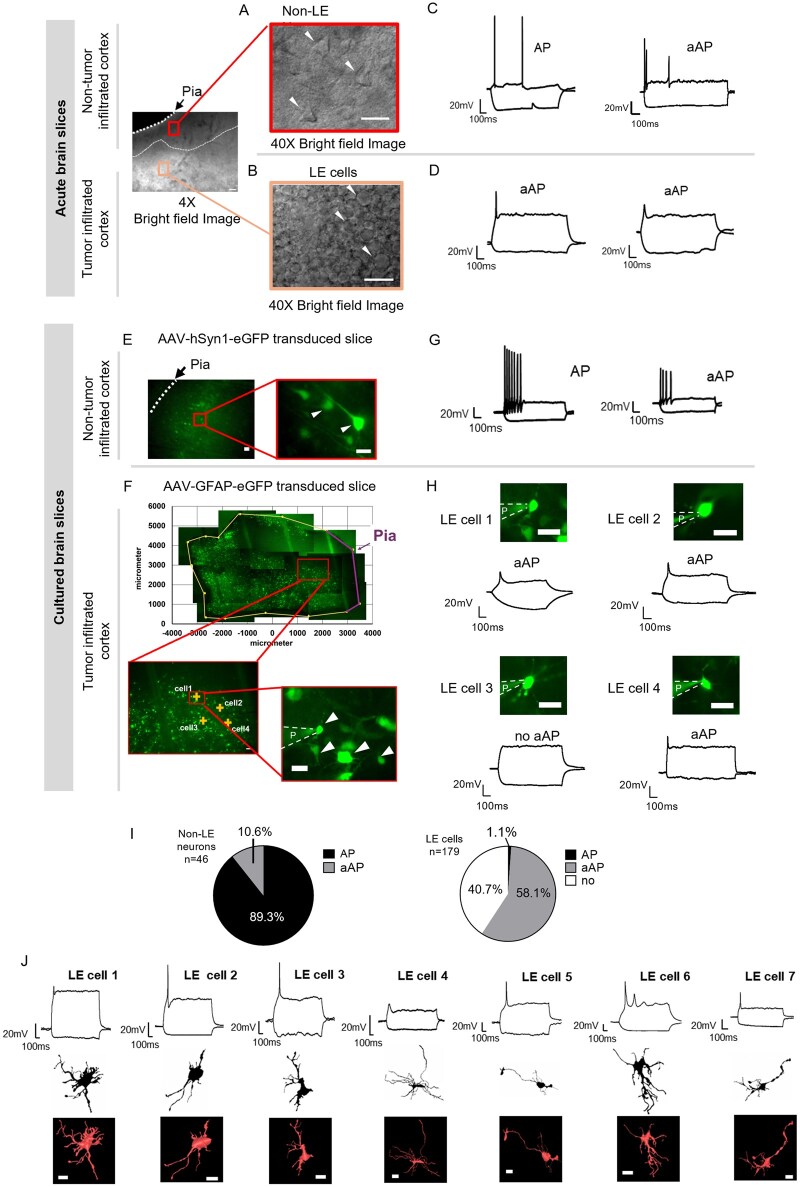
Neocortical tumor leading edge (LE) cells exhibit aberrant action potentials (aAPs). Left upper panel: Bright-field image at 4× magnification showing a human tumor-infiltrated neocortical brain slice. The white dashed line delineates the boundary between preserved cortex (above) and tumor-infiltrated area (below). In the 4× magnification image, the scale bar corresponds to 100 µm. In the 40× magnification image, the scale bar represents 20 µm. (A) High-magnification (40×) bright-field image of preserved neocortex (red inset) reveals pyramidal non-LE neurons with characteristic triangular somata (white arrowheads). (B) High-magnification (40×) bright-field image of the tumor LE cells (orange inset) shows dense clusters of small, round cells and disrupted laminar organization (white arrowheads). (C) Representative whole-cell patch-clamp recordings of neurons from acute slices showing one typical evoked action potential (AP) and one aAP upon depolarizing current injection, along with their corresponding membrane responses upon hyperpolarizing current injection. (D) Representative whole-cell patch-clamp recordings from LE cells showing aAPs upon depolarization and the corresponding membrane response upon hyperpolarization. (E) Representative images from cultured brain slices show AAV-hSyn-eGFP labeling, enabling visualization of non-LE neurons (white arrowheads) in the preserved neocortex. The red box indicates the region shown at higher magnification in the corresponding zoomed-in insets. (F) AAV-GFAP-eGFP labeling identified putative GBCs and glial cells at the tumor leading edge. Red boxes mark regions of interest in the low-magnification images, which are shown at higher magnification in the corresponding zoomed-in insets. AAV-GFAP-eGFP-labeled LE cells are indicated by white arrowheads, and the patch pipette (P) is outlined with a white dashed line. (G) Representative whole-cell patch-clamp recordings of AAV-hSyn-EGFP-labeled neurons in slice culture showed typical APs, with a minority exhibiting aAPs upon depolarizing current injection, presented along with their membrane response upon hyperpolarizing current injection. (H) Representative images show LE tumor cells 1-4, recorded from the neocortical tumor LE, exhibiting either aAPs or no excitability. Images show the patched LE cells and the patch pipette (P), outlined with white dashed lines. (I) Pie charts summarizing electrophysiological characteristics of all patched cells: 89.3% (42/47) of neocortical non-LE neurons exhibited APs, while 10.6% (5/47) showed aAPs. Among LE cells, 58.1% (104/179) exhibited aAPs, 40.7% (73/179) were non-excitable, and 1.1% (2/179) exhibited APs. (J) Morphology reconstruction of 7 recorded LE cells. Representative membrane responses to a 1-s hyperpolarizing current step and a suprathreshold depolarizing current step. Each reconstruction illustrates the somatodendritic architecture of 7 individual cells recorded from the neocortical glioblastoma LE. Matched traced cell morphologies are shown in the middle row for recorded LE cells. Scale bar: 20 µm.

To investigate electrophysiological and morphological properties, we performed whole-cell patch-clamp recordings targeting LE cells and non-LE neurons in the adjacent non-tumor-infiltrated neocortex. The LE was defined as the region infiltrated by tumor cells, located immediately superficial to the dense tumor core and adjacent to the structurally intact, preserved neocortex (Figures 1D and 2A and Supplementary Figure S2). Pyramidal neurons located outside the tumor LE, referred to as non-LE neurons, were identified under bright-field microscopy at 40× magnification based on their characteristic triangular-shaped somata, measuring approximately 20–50 µm in diameter (Figure 2A, red inset).^32^ In contrast, LE cells formed dense clusters within regions of disrupted cortical lamination and displayed relatively round somata, typically measuring 10–20 µm^33^ (Figure 2B, orange inset). The non-LE neurons in acute slices displayed normal overshooting APs (Figure 2C). Intriguingly, we found that 59.6% (28 out of 47) of LE cells exhibited aAPs when depolarized by a positive current injection (Figure 2D). AP and aAP differed in waveform features such as amplitude, half-width, maximum rise slope, and minimum decay slope (Supplementary Figure S3A), as shown in a 2D plot of amplitude and decay slope (Supplementary Figure S3),^26^ followed by a hierarchical clustering analysis (Supplementary Figure S3C).

To target cells of putative glial and neuronal lineage, we adopted an ex vivo slice culture paradigm combined with AAV-mediated enhancer-based fluorescent labeling.^23–25^ We used AAV1-hSyn1-EGFP-WPRE-hGHp(A) to label cells of putative neuronal lineage via the human synapsin-1 promoter (hSyn1), a conventional marker of neuronal identity^34^ (Figure 2E and red inset), and AAV5-hGFAP-hHEbl/E-EGFP-bGHp(A) to label GFAP-expressing cells of putative glial lineage (Figure 2F and red insets). hSyn1-eGFP⁺ cells in structurally preserved neocortex had soma sizes and shapes equivalent to pyramidal neurons, and evoked APs with corresponding waveforms (Figure 2E and G). In contrast, GFAP-eGFP⁺ cells in the LE appeared as dense clusters of small, round cells within disrupted cortical structure (Figure 2F and H). We performed additional staining and found that the LE GFAP-eGFP⁺ cells show 49% and 71% colocalization with Olig2 and Nestin, respectively, suggesting the labeled cells were unlikely to be astrocytes (Supplementary Figure S4).

The electrophysiological properties were examined by a standardized protocol testing active and passive membrane properties.^35^ From all recorded cells in both acute and cultured brain slices (documented in Supplementary Figure S5), we observed that non-LE neurons were excitable upon positive current injections. Most non-LE neurons generated normal APs (89.3%, 42 out of 47 cells), with only 5 non-LE neurons exhibiting aAPs (10.7%, Figure 2I and J). Intriguingly, 58.1% (104 out of 179 cells) of all recorded LE cells exhibited active membrane properties upon depolarization (Figure 2H), whereas 40.7% (73 out of 179 cells) were non-excitable (Figure 2I). Only a small subset (1.1%, 2 out of 179) of LE cells displayed normal APs (Figure 2I). Morphological analysis of 7 patched LE GFAP-eGFP⁺ cells filled with Alexa594 and biocytin revealed structurally abnormal somatodendritic features, showing irregular processes (Figure 2J). Collectively, these results demonstrate that the majority of LE cells exhibit neuronal-like excitability, characterized by the presence of aAPs, accompanied by morphological alterations.

### Distinct Membrane Properties and Spontaneous Excitatory Input Strength between Neocortical Non-LE Neurons and LE Cells

**Figure 3.**
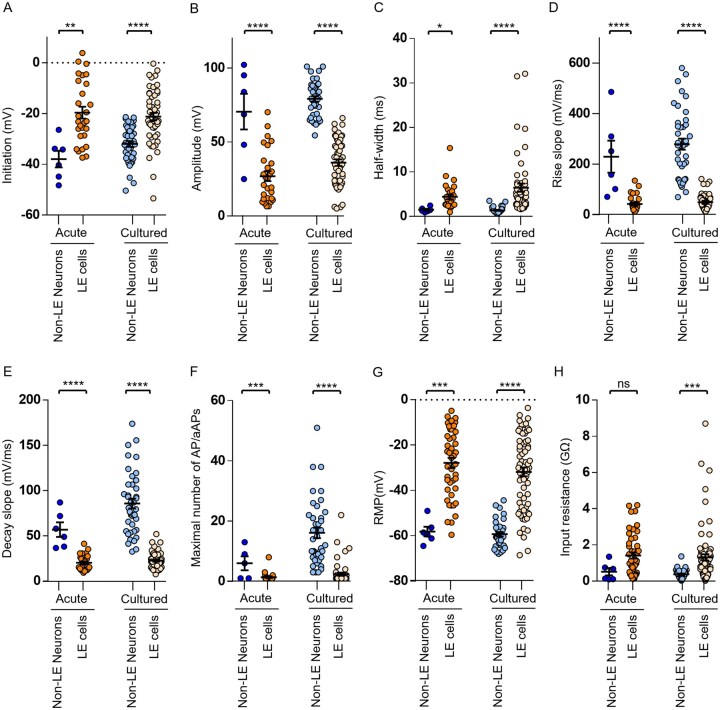
Electrophysiological characteristics of aberrant action potentials (aAPs)/action potentials (APs) in leading edge (LE) cells and neocortical non-LE neurons. (A-H) Quantitative comparisons of APs/aAPs waveform parameters and passive membrane properties, including resting membrane potential (RMP) and input resistance, between non-LE neurons and LE cells recorded from acute brain slices. Boxplots show (A) initiation potential, (B) amplitude, (C) half-width, (D) rise slope, (E) decay slope, (F) maximal number of AP/aAP, (G) RMP, and (H) input resistance. In acute brain slices, non-LE neurons (*n* = 6) exhibited significantly lower initiation potential (−37.95 ± 3.27 mV), higher amplitude (70.51 ± 12.05 mV), shorter half-width (1.51 ± 0.22 ms), faster rise slope (230.2 ± 63.03 mV/ms), faster decay slope (56.72 ± 8.07 mV/ms) and generated more APs/aAPs (6 ± 2.32) compared to LE cells (*n* = 28) (initiation: −19.65 ± 2.35 mV, amplitude: 26.97 ± 3.38 mV, half-width: 4.40 ± 0.55 ms, rise slope: 42.63 ± 6.17 mV/ms, decay slope: 20.23 ± 1.55 mV/ms, maximal number of AP/aAP: 1.39 ± 0.26). Non-LE neurons displayed a more hyperpolarized RMP of −58.20 ± 2.14 mV and lower membrane input resistance of 0.52 ± 0.20 GΩ compared to LE cells (RMP: −27.84 ± 2.23 mV, input resistance: 1.41 ± 0.17 GΩ). In cultured slices, non-LE neurons (*n* = 40) showed less depolarized initiation potential (−31.96 ± 1.15 mV), higher amplitude (79.14 ± 1.87 mV), shorter half-width (1.36 ± 0.11 ms), faster rise slope (279.7 ± 21.60 mV/ms), faster decay slope (85.65 ± 5.49 mV/ms) and generated more APs/aAPs (16.10 ± 1.73) than LE cells (*n* = 52) (initiation: −21.19 ± 1.46 mV, amplitude: 36.10 ± 2.21 mV, half-width: 6.46 ± 1.05 ms, rise slope: 49.87 ± 4.30 mV/ms, decay slope: 22.58 ± 1.26 mV/ms, maximal number of AP/aAP: 2.39 ± 0.53). In cultured slices, non-LE neurons displayed a more hyperpolarized RMP of −59.34 ± 0.94 mV and lower membrane input resistance of 0.37 ± 0.04 GΩ than in LE cells (RMP: −31.88 ± 1.93 mV, input resistance: 1.30 ± 0.18 GΩ). Statistical significance: *P < .05, **P < .01, ***P < .005, ****P < .001.

We compared the active membrane properties of non-LE neurons and LE cells in acute and cultured slices. Six AP/aAP features were measured, including: initiation (threshold, mV), amplitude (mV), half-width, maximum rise slope (mV/ms), maximum decay slope (mV/ms), and maximum firing rate.^36^ We found that LE cells exhibited a more depolarized threshold to initiate an aAP compared to non-LE neurons (Figure 3A). The aAP generated by LE cells had smaller amplitude, broader half-width, and slower rise and decay slopes compared to non-LE neurons (Figure 3B-E). Furthermore, LE cells fired fewer aAPs compared to non-LE neurons (Figure 3F). These features are reminiscent of immature neurons or early-stage stem cell-derived neurons.^37^ We also evaluated the consistency of active membrane properties over time in culture for both non-LE neurons and LE cells, and there was no significant trend of alteration of most electrophysiological features with increasing culture days (Supplementary Figure S6). In addition to membrane property measurements, in a subset of experiments, we recorded spontaneous excitatory postsynaptic current (sEPSC) from 40% (6/15) of tested non-LE neurons (Supplementary Figure S7A) and 11% (7/62) of tested LE cells (Supplementary Figure S7B). Non-LE neurons received more frequent sEPSC compared to LE cells, while LE cells showed faster sEPSC kinetics, with faster sEPSC onset and shorter half-width compared to non-LE neurons (Supplementary Figure S7C-H).

In terms of passive membrane properties, the RMP of LE cells was significantly depolarized compared to that of non-LE neurons (Figure 3G). In addition, the input resistance (*R*_in_) of the LE cells was higher compared to non-LE neurons (Figure 3H). The RMP and *R*_in_ of all groups had no significant correlation with days in vitro, indicating stable passive membrane properties over the culture period (Supplementary Figure S6). These unique characteristics of LE cells, with depolarized RMP and high *R*_in_, mimic those of hybrid cells (HCs) in IDH-mutant gliomas and set them apart from astrocytes, which typically have a hyperpolarized RMP, lower membrane resistance, and are unable to generate aAP/APs.^38^

### aAPs Generation in LE Cells Requires Na_V_ and K_V_ Channels

**Figure 4.**
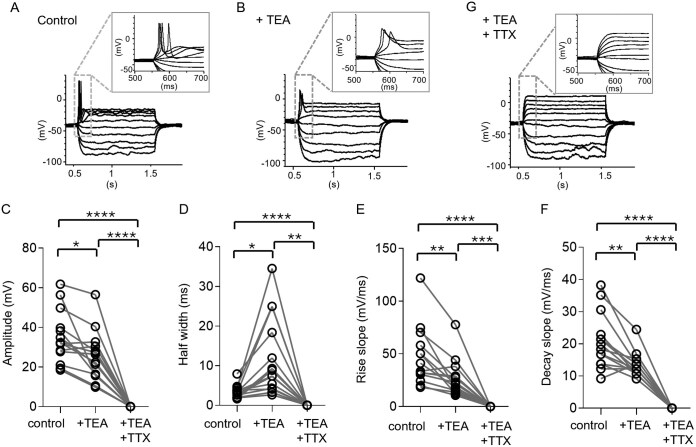
Contribution of Na_V_ and K_V_ channels to aberrant action potential (aAP) generation. (A) Illustrative aAPs induced by a 1-s square current injection ladder in an leading edge (LE) cell. The gray-dashed box indicates the first 200 ms during which the aAP occurs. (B) Illustrations presenting changes in aAP morphology upon application of the K_V_ blocker TEA, highlighting alterations compared to the control condition. (C) Comparison of amplitude parameter for 14 TME cells under 3 conditions: Control (35.13 ± 3.53 mV), TEA (25.32 ± 3.34 mV), and TEA+TTX (0 mV), exhibiting a reduction in aAP amplitude in the presence of TEA and a complete absence in the combined presence of TEA and TTX. TTX is a Na_V_ channel blocker. (D) Comparison of half-width parameter under 3 conditions: Control (3.39 ± 0.43 ms), TEA (11.93 ± 2.64 ms), and TEA+TTX (0 ms), exhibiting a significantly wider half-width in the presence of TEA (*P < .05) and a complete absence in the combined presence of TEA and TTX. (E) Comparison of rise slope parameter under 3 conditions: Control (46.97 ± 7.44 mV/ms), TEA (26.80 ± 4.70 mV/ms), and TEA+TTX (0 mV/ms), exhibiting a significant reduction in the presence of TEA and a complete absence in the combined presence of TEA and TTX. (F) Comparison of decay slope parameter under 3 conditions: Control (20.77 ± 2.14 mV/ms), TEA (13.34 ± 0.94 mV/ms), and TEA+TTX (0 mV/ms), exhibiting a significant reduction in the presence of TEA and a complete absence in the combined presence of TEA and TTX. Comparative analysis among paired data from 3 groups was performed using 2-way ANOVA followed by Tukey’s multiple comparison test. (G) Absence of aAP induction upon depolarization after the combined application of both TEA and TTX. Statistical significance: *P < .05, **P < .01, ***P < .005, ****P < .001.

To test if the ion channels required for LE cell aAP generation were similar to those of non-LE neurons, we first applied tetraethylammonium (TEA, 30 mM) to block voltage-gated potassium channels in general. This resulted in a notable decrease in the aAP amplitude compared with baseline (Figure 4A-C). Additionally, we observed an increase in aAP half-width (Figure 4D) along with slower rise and decay slopes (Figure 4E and F). Subsequent bath application of tetrodotoxin (TTX, 1 mM), a voltage-gated sodium channel blocker, eliminated the aAP (Figure 4G). These findings elucidate the cellular components involved in LE cell aAPs and further confirm that the electrical excitability of neocortical LE cells resembles that of immature neurons and HCs in IDH-mutant gliomas.

### Heterogeneous Cellular States of LE Cells and Distinct Signaling in GBCs by Patch-Seq

**Figure 5.**
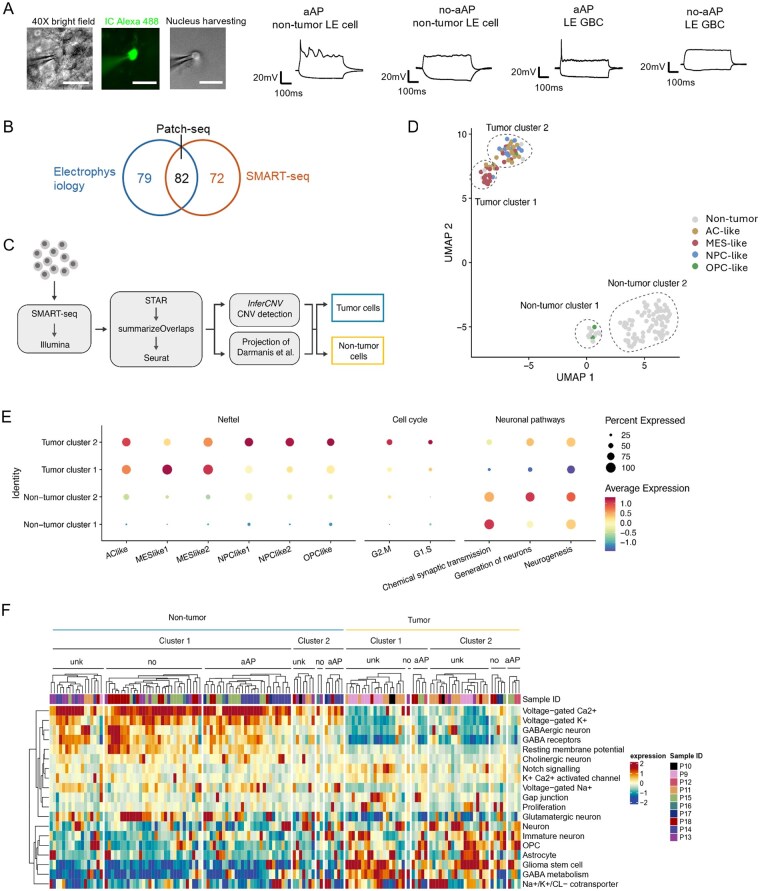
Characterization of aberrant action potential (aAP) cells using Patch-seq and analysis workflow. (A) Representative images of one cell that underwent the Patch-seq procedure (left), recorded with an intracellular solution containing Alexa 488 (middle). After electrophysiological recording, the nucleus was extracted (right) and collected for SMART-seq, followed by transcriptomic analysis. Representative electrophysiological traces from nuclei harvested from the neocortical tumor leading edge (LE), including an aAP non-tumor cell, a no-aAP non-tumor cell, an aAP GBC, and a no-aAP GBC (1-s depolarization and hyperpolarization current injection). Scale bars: 20 µm. (B) Venn diagram illustrating the total number of cells included in electrophysiological and/or transcriptomic analysis. Out of 233 cells, 79 were analyzed only by electrophysiology, 82 had both electrophysiological and transcriptomic parameters measured, and 72 were collected only for transcriptomic analysis. (C) Workflow of Patch-seq data generation and cell characterization. The single cells were processed using the SMART-seq protocol, followed by the identification of tumor cells and subsequent transcriptomic analysis. The resulting reads were aligned using STAR, counted using summarizeOverlaps, and imported into Seurat for downstream analysis. Cell type annotation was performed using a combination of InferCNV and cell type projection. (D) UMAP visualization of cells, colored by their cellular state. The distinct tumor and non-tumor clusters are highlighted. (E) A Dot plot showing the average expression of cellular state signatures, cell cycle, and neuronal pathways that were divided into 4 clusters. (F) Heatmap of pathway expression across single cells.

To determine the cell types and molecular profiling of neocortical LE cells, we performed Patch-seq on acute and slice culture configurations (Figures 1E, 1H, and 5A; detailed cell information in Supplementary Table S1).^25^ Collectively, a major subset (144/154) of neocortical LE cells from 10 patients had sufficient cDNA for further transcriptomic analysis, and 82 of these cells had electrophysiological parameters measured (Figure 5B). We identified neoplastic GBCs within the LE cell population using a combination of label-transfer from a comparable dataset of glioblastoma tissue sampled at the tumor core and peripheral tissue,^28^ and iterative inferring copy-number variant (CNV) analysis, validated using shallow whole genome sequencing of the matched patient material^39^ (InferCNV; Figure 5C; Supplementary Figure S8; see Methods). Transcriptomic profiling revealed 4 distinct LE cell clusters, visualized in the 2D UMAP embedding (Figure 5D). Two clusters of neocortical LE cells (53/144 LE cells) were classified as neoplastic GBCs (Tumor clusters 1 and 2; Figure 5D). In addition, the 2 clusters of non-tumor cells (non-tumor clusters 1 and 2) exhibit enrichment of neuronal pathways compared to GBCs, but slightly enriched astrocytic signatures. These cells were referred to as non-tumor cells (Darmanis dataset^28^; Figure 5E; Supplementary Figure S9A).

We next mapped the GBCs to the widely used cellular states defined by Neftel et al.^4^ The cellular composition was heterogeneous: one cluster predominantly consisted of MES-like cells (Tumor cluster 1, Figure 5D), while the other contained mainly AC-like and NPC-like cells (Tumor cluster 2, Figure 5D). Only 5 cells showed an OPC-like state (Figure 5A). Tumor cells in cluster 2 exhibited elevated levels of G1/S and G2/M gene expression (Figure 5E), suggesting that these cells are actively dividing. In contrast, the non-tumor cells showed low expression of tumor cellular state markers (Figure 5E), as expected.

A total of 15 out of 53 GBCs were electrophysiologically recorded: 9 GBCs showed aAPs, while 6 GBCs did not (no-aAP). Of the 9 GBCs, 2 were classified as MES-like, 5 as AC-like, and 2 as OPC-like cell states. aAP GBCs were detected across patients and under different conditions (Supplementary Figure S9B). On the other hand, 67 out of 91 non-tumor cells were electrophysiologically recorded, with 34 showing aAP and 33 no-aAP. The fraction of non-tumor cells and GBCs was similar under both acute and cultured slice configurations (acute slice: 25 non-tumor cells, 5 GBCs; cultured slice: 42 non-tumor cells, 10 GBCs). We found no significant differences when comparing the electrophysiological properties of GBCs and non-tumor cells (Supplementary Figure S10). In addition, we detected spontaneous aAP-like transient depolarizations without current injection in a small subset of aAP GBCs and non-tumor cells, despite their varied spontaneous activity (Supplementary Figure S11). In contrast, transcriptomic profiling revealed that non-tumor cells exhibited a higher expression of Na_V_, K_V_, and Ca_V_ ion channel signaling, compared to GBCs, as well as elevated expression of GPC3, encoding the synaptogenic molecule glypican-3, also known to regulate apoptosis and facilitate gliomagenesis and hyperexcitability in glioblastoma^40^ (Figure 5F, Supplementary Figures S12 and S13A-D). On the other hand, compared to non-tumor cells, GBCs exhibited enriched immature neuron and proliferation signaling, as well as other glial cell marker expressions, and an increased G1-S phase transition-regulating gene CCND2 (Figure 5F, Supplementary Figure S13E). In addition, GBCs showed enriched GABA metabolism and chloride homeostasis signaling compared to non-tumor cells, both related to the GABAergic transmission regulation (Figure 5F, Supplementary Figure S12).

**Figure 6.**
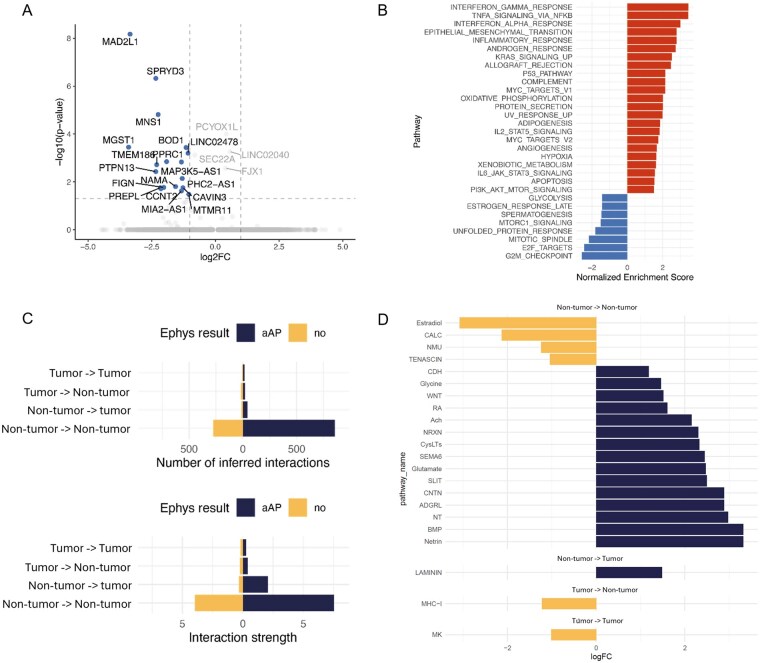
Transcriptomic and intercellular signaling correlate with aberrant action potential (aAP) generation in leading edge (LE) cells. (A) Volcano plot comparing gene expression between aAP GBCs and no-AP GBCs. (B) Bar plot showing the result of GSEA between the same groups as A. (C) Bar plot showing cell-cell interaction results: (left) number of interactions, and (right) interaction strength. (D) Bar plot shows differential pathway signaling probabilities between aAP and no-aAP cells, shown as log2 fold-change (logFC). For each pathway, signaling probabilities and associated *P* value were computed separately for aAP and no-aAP cells. Positive logFC values (blue) indicate higher signaling probability in aAP cells, whereas negative values (orange) indicate higher signaling probability in no-aAP cells.

We next investigated the cellular states of electrically distinct GBCs. Overall, GBCs exhibited high cell-state heterogeneity in both acute and cultured slices, a phenomenon observed across patients, with a slight enrichment for the AC-like cell state (Supplementary Figure S9B-E, SupplementaryTable S2).^4^ We further performed differential gene expression analysis followed by GSEA on the ranked gene lists between aAP and no-aAP GBCs (Figure 6A and B). aAP GBCs showed reduced proliferation-related signaling pathways, but increased inflammatory/immune response, mesenchymal transition, hypoxia, and p53 signaling compared to no-aAP GBCs (Figure 6B). We identified genes enriched in aAP and no-aAP GBCs and quantified their enrichment in the GLASS dataset to test survival correlations. We found a trend but insignificant correlation between the aAP GBC gene signature and enhanced survival (Supplementary Figure S13F). In terms of ion channel signaling, aAP GBCs showed a trend of higher expression of Na_V_ channel SCN3A compared to no-aAP GBCs, supporting their difference in aAP generation capability (Supplementary Figure S13G). Interestingly, aAP-negative GBCs showed significantly higher expression of L-type voltage-gated calcium channel CACNA1C (Ca_V_1.2) compared to aAP GBCs (Supplementary Figure S13I). We also investigated the expression of thrombospondin-1 (THBS1), which is involved in brain.^8^ Two aAP GBCs showed significantly higher THBS1 expression (2/9 cells), while no-aAP GBCs showed lower THBS1 expression (6/6 cells, Supplementary Figure S13L).

Lastly, we used CellChat to quantify matched ligand–receptor expressions between LE cells. We observed a higher number of inferred interactions and higher interaction strength between aAP cells than between no-aAP cells (non-tumor-to-non-tumor and non-tumor-to-GBCs interactions, Figure 6C). We further extracted the CellChat analysis on a pathway basis. In non-tumor-to-non-tumor interaction, aAP cells showed higher glutamate, glycine, and acetylcholine signaling probability compared to no-aAP cells, along with higher probability in synaptic contact pathways, such as ADGRL and NRXN (Figure 6D), consistent with our findings that non-tumor cells have enriched neuronal transcriptomic signatures. Conversely, there was a reduced probability of estradiol and calcium-related signaling (CALC, NMU) and extracellular matrix signaling (TENSCIN, Figure 6D). We also identified increased LAMININ probability (non-tumor-to-tumor), reduced MHC-1 (tumor-to-non-tumor), and reduced midkine (MK) signaling probabilities (tumor-to-tumor) in aAP cells compared to no-aAP cells (Figure 6D).

In conclusion, patch-seq and transcriptomic analysis confirmed that GBCs can generate aAPs with features similar to those of non-tumor cells. However, several voltage-gated ion channel genes were more highly expressed in non-tumor cells. GBCs exhibited heterogeneous cellular states, and cells across different states displayed the capacity to generate aAPs, suggesting a shared feature across different GBC populations at the tumor LE. In addition, no-aAP GBCs were enriched for mitotic signaling, whereas aAP GBCs showed reduced proliferation signaling and increased inflammatory pathways. aAP cells generally displayed stronger cell-to-cell interactions than no-aAP cells at the LE, with a higher probability of neurotransmission and synaptic contact signaling.

## Discussion

Glioblastoma is an incurable brain cancer characterized by significant cellular heterogeneity. Using human acute slices and slice cultures, combined with single-cell whole-cell patch-clamp recordings and patch-seq, we provide robust evidence for electrophysiologically active GBCs in the cortical tumor LE. Moreover, GBCs and non-tumor cells exhibited strikingly similar electrophysiological properties, including aAP phenotype and features of hyperexcitability. Collectively, the morpho-electric features of LE cells paralleled recent descriptions of HCs in IDH-mutant glioma.^19^ Accordingly, transcriptomic signatures of non-tumor cells were predominantly enriched in neuronal but also minor astrocytic features, while the aAP phenotype were observed across diverse GBC states.

We found that about 58% of LE cells, including GBCs and non-tumor cells, generated similar aAPs via voltage-gated Na^+^ and K^+^ channels as neuronal APs. aAP LE cells demonstrated stronger ligand-receptor interactions, and GBCs exhibited enrichment of mitotic signaling pathways compared to non-tumor cells. Despite their distinct molecular profiles, GBCs and non-tumor cells converged on a shared electrophysiological phenotype with depolarized RMP, elevated input resistance, and a tendency to fire single aAPs, unlike neurons outside the LE. These features align with reports of glioma-induced cortical hyperexcitability and suggest microenvironmental changes at the neocortical LE, affecting both tumor and non-tumor cells. Literature highlights multiple glioma-dependent mechanisms contributing to hyperexcitability,^10^^,^^41–44^ including elevated extracellular glutamate^42^^,^^43^ and secretion of synaptogenic molecules, such as glypican-3.^40^ While we did not dissect these mechanisms experimentally, they align with our CellChat-based inference of increased signaling probability for glutamate (Figure 6D) and the elevated GPC3 expression in non-tumor cells (Supplementary Figure S13D), potentially contributing to alterations of the tumor microenvironment at the LE.

GSEA revealed that aAP GBCs were enriched in inflammatory/immune, hypoxic, angiogenic, and p53 pathways, collectively indicating a multifaceted contribution across diverse hallmarks of cancer. LAMININ signaling between aAP cells observed in CellChat analysis further indicates a potential non-tumor-to-tumor contribution via extracellular matrix-driven malignancy mechanisms. Conversely, reduced MHC-1 signaling suggested tumor cell resistance to immune regulation, while reduced MK signaling was consistent with reduced enrichment of proliferation-related pathways observed in aAP GBCs by GSEA. Finally, elevated enrichment in mesenchymal transition pathways indicates that the aAP phenotype may be associated with the integration of GBCs into the multicellular network of MES-like GBCs at the LE.^45^

Spikelet-like aAP phenotypes have previously been observed in neurons and certain glial populations under physiological conditions.^46^^,^^47^ Neuronal spikelets can arise from distal axonal action potentials, dendritic spikes, gap junctions, or ephaptic mechanisms,^48^ and may influence neuronal synchronization and synaptic plasticity.^48^ In glia cells, NG2-positive OPCs can exhibit similar aAPs in the developing or injured nervous system.^46^^,^^47^ These precedents raise the possibility that aAPs in LE cells may reflect specialized functions related to active release mechanisms, invasion behavior, or local network integration,^16^^,^^42^^,^^43^ as proposed for GABAergic OPCs and hybrid glial/NPC cells in IDH-mutant glioma.^19^ Our work further complements recent spatial transcriptomic studies, revealing a pattern of neurodevelopmental pathways and glial cell differentiation at the human glioblastoma infiltration zone with heterogeneous cell states (mainly NPC-, OPC-, and AC-like states),^33^^,^^49^ with increased expression of synaptic gene programs.^50^ The uniformity of electrophysiology properties across distinct GBC states and non-malignant LE cells observed is possibly specific to the neocortical niche. Moreover, aAP generation across cell types may facilitate network communication with maximized information encoding and processing capacity by distributing function across diverse cells, an organizing principle extensively described in neuronal networks. The cellular and functional diversity at the LE may provide glioblastoma with high flexibility in shaping invasion strategy, proliferative dynamics, and interactions with the surrounding neural circuitry.

Aberrant action potential generation has rarely been observed in cultured tumor cells or xenograft glioblastoma,^9^ possibly due to loss of native microenvironmental structure. In contrast, 2 studies from the 1990s reported aberrant excitability of putative GBCs,^51^^,^^52^ preserving the native cytoarchitecture, tumor-neuron interactions, and the tumor microenvironment, although a recent study by Tetzlaff et al.^13^ Here, we also used human glioblastoma slices, but specifically targeted cells at the glioblastoma-neocortex interface, expectedly enriched in neuronal cell types, and therefore our results may be specific to this niche.

Our findings resemble the electrophysiological features of HCs described by Curry et al. (2024) in IDH-mutant glioma.^19^ Although the anatomical origin of the sample specimens was not specified in that study, we demonstrate that aAP GBCs and non-tumor HCs with predominant neuronal and minor astrocytic features reside at the cortical LE across GBM patients. Here, we found that aAP GBCs spanned AC-like, MES-like, and OPC-like states. While GBCs in our study generally had elevated GABA metabolism pathways, they did not exhibit enrichment in specific neuronal subtype signatures, including GABAergic signatures. This may indicate broader heterogeneity compared to IDH-mutant gliomas, in which spiking cells were annotated as GABAergic- and OPC-like (comparative findings summarized in Supplementary Table S3). Together, these results suggest that aAP generation may represent a generalizable pathophysiological feature of cells in both IDH-mutant and IDH-wildtype gliomas.

### Future Perspectives

This study identifies electrically active GBCs and non-tumor cells as a consistent feature of human glioblastoma and reveals convergence towards a shared electrophysiological phenotype despite molecular heterogeneity. Future studies are needed to clarify the underlying mechanisms and functional significance of these findings across glioma subtypes and detail their involvement in tumor progression, invasion, and resilience. Our interpretations based on the GSEA and CellChat analyses are hypothesis-generating and need to be studied further, including our findings of reduced mitotic pathway activity and elevated inflammatory/immune, angiogenic, and mesenchymal transition pathway activity in aAP GBCs, Ca_V_1.2 channel enrichment in no-aAP GBCs, and the impact of CCND2 and GPC3 on GBC progression. SCRAM-based profiling of LE cells would be relevant to form a direct comparison of cell identity of aAP GBCs in IDH-mutant and IDH-wildtype glioma.^19^ Moreover, investigations are needed to elucidate the mechanisms underlying altered physiological properties of non-tumor cells, the impact of aAP LE cells on the pathogenesis, and the possible influence of non-LE neurons as oncogenic modulators of the LE microenvironment.

### Limitations of the Study

This study included a limited number of patient samples and cell numbers, and future work with larger cohorts will be important to refine LE cell type annotation, connectivity, molecular signaling, and heterogeneity. Our random sampling patch-seq experiment indicates that the AAV-hGFAP-eGFP virus does not label astrocytes at the LE, although HCs with slight GFAP expression may be targeted. Differences in computational annotation may contribute to variations in cell type profiling. In addition, transcripts of voltage-gated ion channels involved in AP generation were detected at low levels from patch-seq nuclei, particularly in GBCs, preventing a statistical separation of aAP and no-aAP GBCs at the transcriptomic level. However, a trend in this direction was observed, and at the protein level, the electrical properties of these cells were distinct in terms of aAP behavior driven by voltage-gated Na^+^ and K^+^ channels. The discrepancy between function and transcriptomic profiling may reflect cross-level comparison (RNA vs protein levels), methodological differences (electrophysiology vs transcriptomics), and technical limitations of mRNA recovery for small-soma cells in patch-seq experiments.

## Supplementary Material

noag059_Supplementary_Data

## Data Availability

The count matrix for the single-nucleus RNA sequencing dataset is uploaded to GEO (GSE315516). The data will be made available upon reasonable request.
