# Supplementary material for: Combined patch-clamp electrophysiology and single-cell genomic analysis reveal spiking tumor cells at the neocortical glioblastoma interface in humans
Source: Neuro Oncol. 2026 Mar 19;28(6):1442–57. doi: 10.1093/neuonc/noag059 (PMC13232509; doi:10.1093/neuonc/noag059)
Supplement: noag059_Supplementary_Data [file noag059_supplementary_data.docx]

**Supplementary Materials**

**Combined Patch-clamp Electrophysiology and Single-Cell Genomic Analysis Reveal Spiking Tumor Cells at the Neocortical Glioblastoma Interface in Humans**

Tong Tong, Josephine D. Hendriksen, Kirstine J. Elbæk, Kata Molnar, Freja V. Christiansen, Attila Ozsvar, Jens T. Eschen, Francisco G. Rodríguez-González, Ilayda D. Pusat, Emilie Littau Christensen, Mads Rahbæk, Kathrine Pii Frederiksen, Søren O. S. Cortnum, Ann K. Sindby, Kaare Meier, Nikola Mikic, Jens C. H. Sørensen, Jonathan T. Ting, Marco Capogna, Bjarne W. Kristensen, Jens R. Nyengaard, Joachim Weischenfeldt, Wen-Hsien Hou*, Anders R. Korshøj*

## Content

[Content 2](#_Toc225452115)

[Supplementary Methods 4](#_Toc225452116)

[DNA extraction 4](#_Toc225452117)

[Correlation between aAP genetic signatures versus survival 5](#_Toc225452118)

[Hematoxylin and Eosin (H&E) staining for tumor area identification 5](#_Toc225452119)

[Antibodies and Morphology Reconstruction 7](#_Toc225452120)

[Supplementary Figures 9](#_Toc225452121)

[Supplementary Figure 1. 9](#_Toc225452122)

[Supplementary Figure 2. 11](#_Toc225452123)

[Supplementary Figure 3. 13](#_Toc225452124)

[Supplementary Figure 4. 14](#_Toc225452125)

[Supplementary Figure 5. 16](#_Toc225452126)

[Supplementary Figure 6. 18](#_Toc225452127)

[Supplementary Figure 7. 20](#_Toc225452128)

[Supplementary Figure 8. 22](#_Toc225452129)

[Supplementary Figure 9. 24](#_Toc225452130)

[Supplementary Figure 10. 26](#_Toc225452131)

[Supplementary Figure 11. 28](#_Toc225452132)

[Supplementary Figure 12. 30](#_Toc225452133)

[Supplementary Figure 13. 32](#_Toc225452134)

[Supplementary Tables 34](#_Toc225452135)

[Supplementary Table 1. 34](#_Toc225452136)

[Supplementary Table 2. 39](#_Toc225452137)

[Supplementary Table 3. 40](#_Toc225452138)

## Supplementary Methods

### DNA extraction

High molecular weight DNA from tumor tissue was extracted using Gentra Purigene Cell kit (Qiagen, 158767) following the manufacturer’s instructions. Briefly, 60 mg of tissue was lysed with 3 ml of Lysis Solution, followed by Proteinase K incubation overnight at 55 °C and RNase treatment for 5 min at 37°C. After 3 min incubation on ice, 100 µl Protein Precipitation Solution was added to the lysate to remove proteins, and then centrifuging for 1 min at 15,000 g. The supernatant was transferred into a clean 1.5 ml microcentrifuge tube with 300 µl isopropanol. DNA was visible as a white pellet after centrifugation. A final wash with 300 µl of 70% ethanol was done to remove remaining impurities. DNA was resuspended in 100 µl DNA Hydration Solution and incubated at 65°C for 1 h to dissolve the DNA. DNA concentration was measured by Qubit dsDNA BR Assay Kit (Thermo Fisher Scientific, Q32850), purity by Denovix spectrophotometer (DS-11Fx) and DNA integrity by TapeStation genomic DNA reagents and ScreenTape assay (Agilent 5067-5366 and 5067-5365, respectively).

**Shallow whole-genome sequencing (sWGS) library preparation and copy number estimation**

Libraries were prepared from 100 ng of genomic DNA using the NEBNext Ultra II FS DNA Library Prep Kit for Illumina (New England Biolabs, E7805S). Briefly, DNA was enzymatically fragmented for 19 min at 37 °C to get fragments averaging 450 bp. After size selection, end repair, A-tailing, and UDI Adaptor Ligation, the final libraries were amplified for 5 PCR cycles and quantified using the Qubit dsDNA High Sensitivity Assay Kit (Thermo Fisher Scientific, Q32851). Size distribution was assessed by Bioanalyzer High Sensitivity DNA Kit (Agilent, 5067-462). Libraries were pooled at 4 nM and sequenced in paired-end mode on a NextSeq 500 using the NextSeq 500/550 Mid-Output v2.5 Kit (Illumina, 20024904), yielding 1X genomic coverage. Copy number variations (CNVs) were inferred from sWGS data using the Absolute Copy number Estimation (ACE) algorithm[^1^](#_ENREF_1).

### Correlation between aAP genetic signatures versus survival

Publicly available GBM bulk RNA-seq data were obtained from the Glioma Longitudinal AnalySiS (GLASS) consortium via Synapse (https://www.synapse.org/glass; data release 2022-05-31). The aAP gene signature was defined as genes with a log2 fold change >1 in aAP GBCs relative to non-aAP GBCs. Each bulk RNA-sequencing IDH-wt GBM sample was scored for aAP signature activity using Gene Set Variation Analysis (GSVA). Samples were classified as aAP-high if their GSVA score exceeded the median value across the cohort. Overall survival was analyzed using Kaplan–Meier curves, and differences between groups were assessed using the log-rank test.

### Hematoxylin and Eosin (H&E) staining for tumor area identification

Adjacent acute tumor-infiltrated neocortical brain slices were fixed in 4% paraformaldehyde (PFA), processed into paraffin blocks. Paraffin blocks were sectioned at 3 µm, mounted on adhesion slides, and stained by H&E. After staining, sections were dehydrated through ethanol (20× dips in 70%, followed by 2 × 5 min in 96%, and 3 × 5 min in 99% EtOH). Clearing was achieved by 2 × 20 dips and 1 × 5 min incubation in xylene. Slides were then mounted with mounting medium and sealed with coverslips (Hounisen). After overnight drying, sections were imaged using a Hamamatsu NanoZoomer (Hamamatsu Photonics; model C9600-12, Japan) at 40x magnification. The tumor leading edge and tumor area were confirmed by alignment with morphologically defined cortical layers in consecutive H&E-stained sections guided by a trained pathologist.

After electrophysiological recordings and cell nuclei harvesting, 350-µm tumor-infiltrated cortical slices were fixed in 4% PFA and paraffin-embedded, then re-sectioned into 10-µm sections and used for histological identification. These slices were transduced with AAV-hGFAP-eGFP, and fluorescence images of the virus-labeled GFAP-eGFP signal were acquired before the slices were processed for immunohistochemistry. Colocalization of the virus-labeled GFAP-eGFP signal with Olig2 and Nestin was assessed on separate slices. Fluorescence images were acquired before staining and compared with the corresponding post-staining images. A total of 200 GFAP-eGFP+ cells were identified in the pre-staining images by matching them to the DAPI signal in the post-staining images. After manual cell matching, colocalization of GFAP-eGFP with Olig2 and Nestin was evaluated separately. Automated cell detection was performed in QuPath-0.6.0-arm64[^2^](#_ENREF_2). Parameters for cell detection were either set to default or modified with sigma=2 and median filter radius=1 px depending on detection performance for each tissue. GFAP-eGFP+/Nestin+ coexpressing cells were identified using QuPath. LE was defined in x40 images based on Gaussian-weighted density maps with LE identified as the area with declining cell density compared with the tumor core in addition to alignment with morphologically evaluated layers in H&E-stained consecutive slides guided by a trained pathologist.

Images were acquired using an Olympus BX50 microscope (Leica Biosystems, Germany) with ×40 magnification. Exposure times were determined using negative-control sections stained with secondary antibodies only to minimize nonspecific signal and were set to 454 ms for DAPI (channel 1), 190 ms for Alexa Fluor 488 (channel 2), and 100 ms for Alexa Fluor 594 (channel 3). H&E images were scanned at ×40 using a Hamamatsu NanoZoomer.

Resliced FFPE slides were placed at 60 °C for 1 hour, followed by deparaffinization in Histolab-Clear (3 × 15 min), and sequential rehydration in absolute, 96%, and 70% ethanol. Slides were rinsed with distilled water and then 0.01 M PBS for 5 minutes. Heat-induced epitope retrieval (HIER) was conducted in a steamer preheated to 99.9 °C using Tris-EDTA buffer (pH 9) for 20 min. Slides were encircled with a PAP pen (Vector Laboratories), washed in PBS-T (PBS with Tween-20) for 5 min, and blocked with 0.2% skim milk in PBS-T for 30 min. Primary antibodies were diluted in PBS-T and applied individually after removing the blocking buffer. Incubation proceeded overnight at 4 °C in a humidified box. The following day, slides were washed in PBS-T (3 × 5 min) and incubated for 2 hours at room temperature in the dark with species-specific secondary antibodies. After washing in PBS, nuclear counterstaining was performed by DAPI (1:10,000, 5 min). Slides were mounted with Fluoromount™ (Sigma-Aldrich), coverslipped, sealed with nail polish, and dried overnight. Prepared slides were stored in light-protected slide boxes at 4 °C before imaging.

### Antibodies and Morphology Reconstruction

Primary antibodies: rabbit anti-NeuN (Sigma-Aldrich, ABN78, 1:1000) and mouse anti-Ki67 (Abcam, ab279653, 1:1000), Rabbit anti-Nestin (Abcam, ab105389, 1:200), Mouse anti-GFAP (Abcam, ab279290, 1:500) Secondary antibodies: donkey anti-rabbit (Abcam, ab150064, 1:500) Alexa Fluor 594 and donkey anti-mouse Alexa Fluor 488 (Abcam, ab150109, 1:500, Abcam). Patched LE cells were reconstructed from stacks of 24–43 images using Neuromantic 1.7.5 software[^3^](#_ENREF_3).
References

**1.** Kuijjer ML, Paulson JN, Salzman P, Ding W, Quackenbush J. Cancer subtype identification using somatic mutation data. *Br J Cancer.* 2018; 118(11):1492-1501.

**2.** Bankhead P, Loughrey MB, Fernández JA, et al. QuPath: Open source software for digital pathology image analysis. *Scientific Reports.* 2017; 7(1):16878.

**3.** Myatt DR, Hadlington T, Ascoli GA, Nasuto SJ. Neuromantic - from semi-manual to semi-automatic reconstruction of neuron morphology. *Front Neuroinform.* 2012; 6:4.

**4.** Donato R, Miljan EA, Hines SJ, et al. Differential development of neuronal physiological responsiveness in two human neural stem cell lines. *BMC Neuroscience.* 2007; 8(1):36.

**5.** Darmanis S, Sloan SA, Croote D, et al. Single-Cell RNA-Seq Analysis of Infiltrating Neoplastic Cells at the Migrating Front of Human Glioblastoma. *Cell Rep.* 2017; 21(5):1399-1410.

## Supplementary Figures

### Supplementary Figure 1.


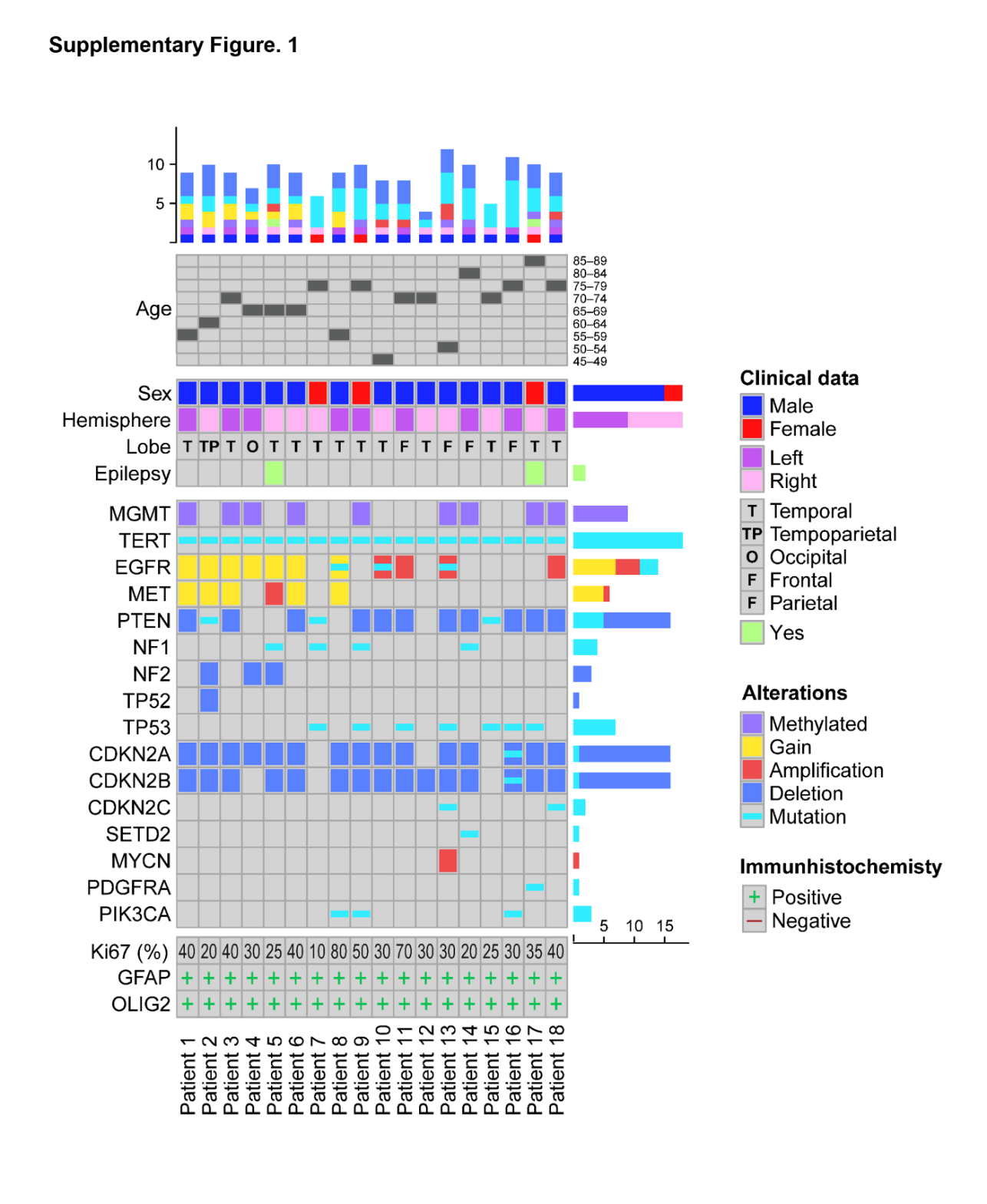


**Supplementary Figure 1.** An OncoPrint plot showing a comprehensive visual representation of both the clinical profiles and genomic landscapes of eighteen glioblastoma patients. At the top of the print, age, sex, tumor location, and epilepsy are shown. In the middle part, an overview of genomic alterations (legend) in particular genes (rows) affecting individual samples (columns) can be seen. Different colors and shapes distinguish the type of alterations. The genes (rows) are sorted based on the frequency of the gene-level alterations in the cohort, as noted on the left of the figure. At the bottom of the print, immunohistochemistry analysis from the pathology reports is recapped. Ki67 labeling is shown as the exact percentage for each patient. The bars at the top of the figure summarize the clinical characteristics and molecular landscape identified in each patient.

### Supplementary Figure 2.


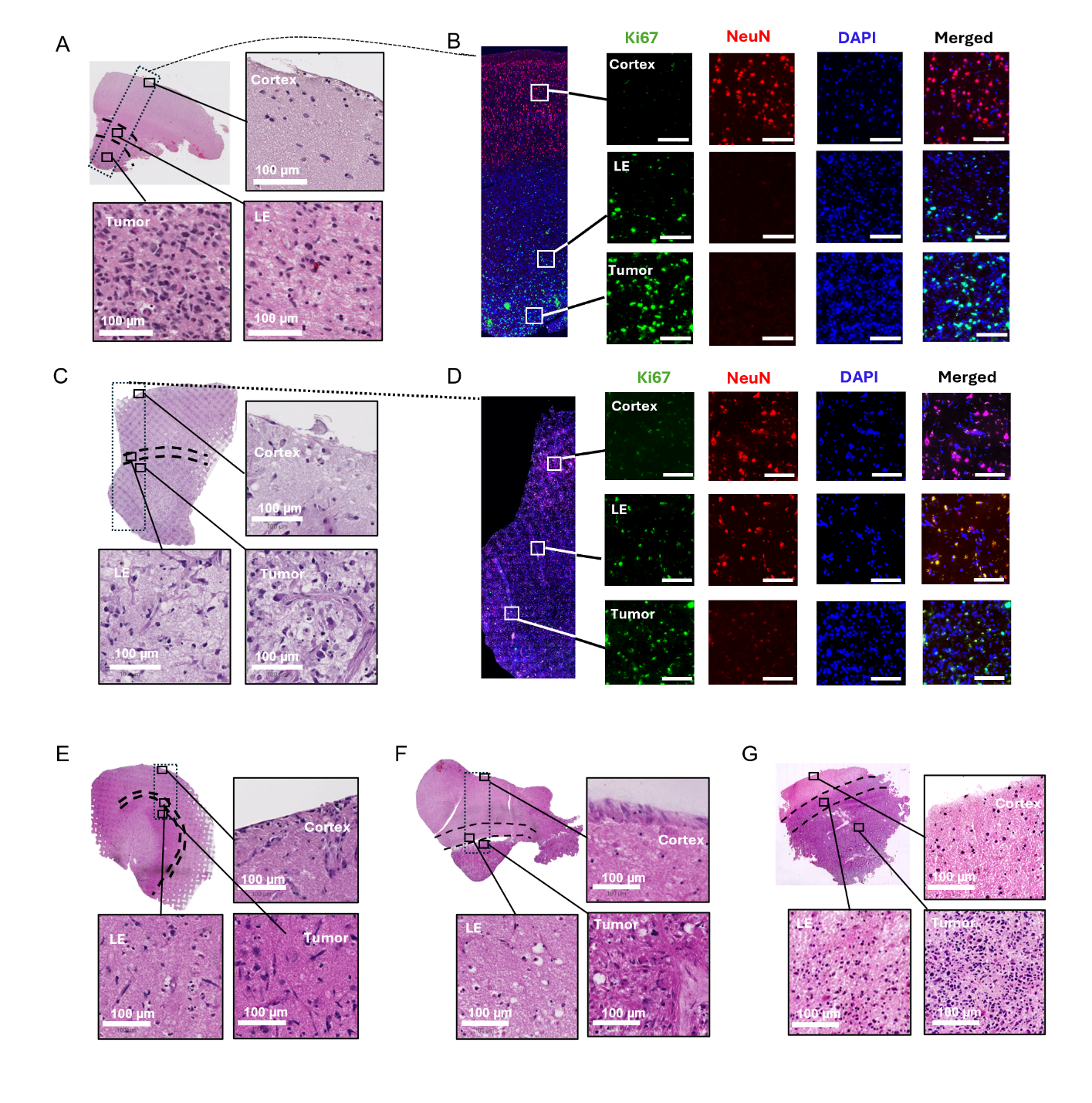


**Supplementary Figure 2. Histological and immunohistochemical identification of the tumor leading edge. A, C, E-G,** Representative H&E-stained sections illustrating the cortical, leading-edge (LE), and tumor regions across multiple patient samples. Low-magnification overview images indicate the approximate location of the LE (dashed lines), with boxed regions shown at higher magnification to highlight cortical area, LE transition zones, and tumor-dense areas. Scale bars are indicated in each panel. **B and D**, Corresponding immunohistochemical staining for Ki67 (green), NeuN (red), and DAPI (blue) in cortex, LE, and tumor regions. Boxed regions in the low-magnification images indicate areas shown at higher magnification in the individual channels and merged images. Ki67 labels proliferative cells, NeuN marks neuronal populations, and DAPI labels cell nuclei. Scale bars in the higher-magnification immunohistochemistry images are 20 µm.

### Supplementary Figure 3.


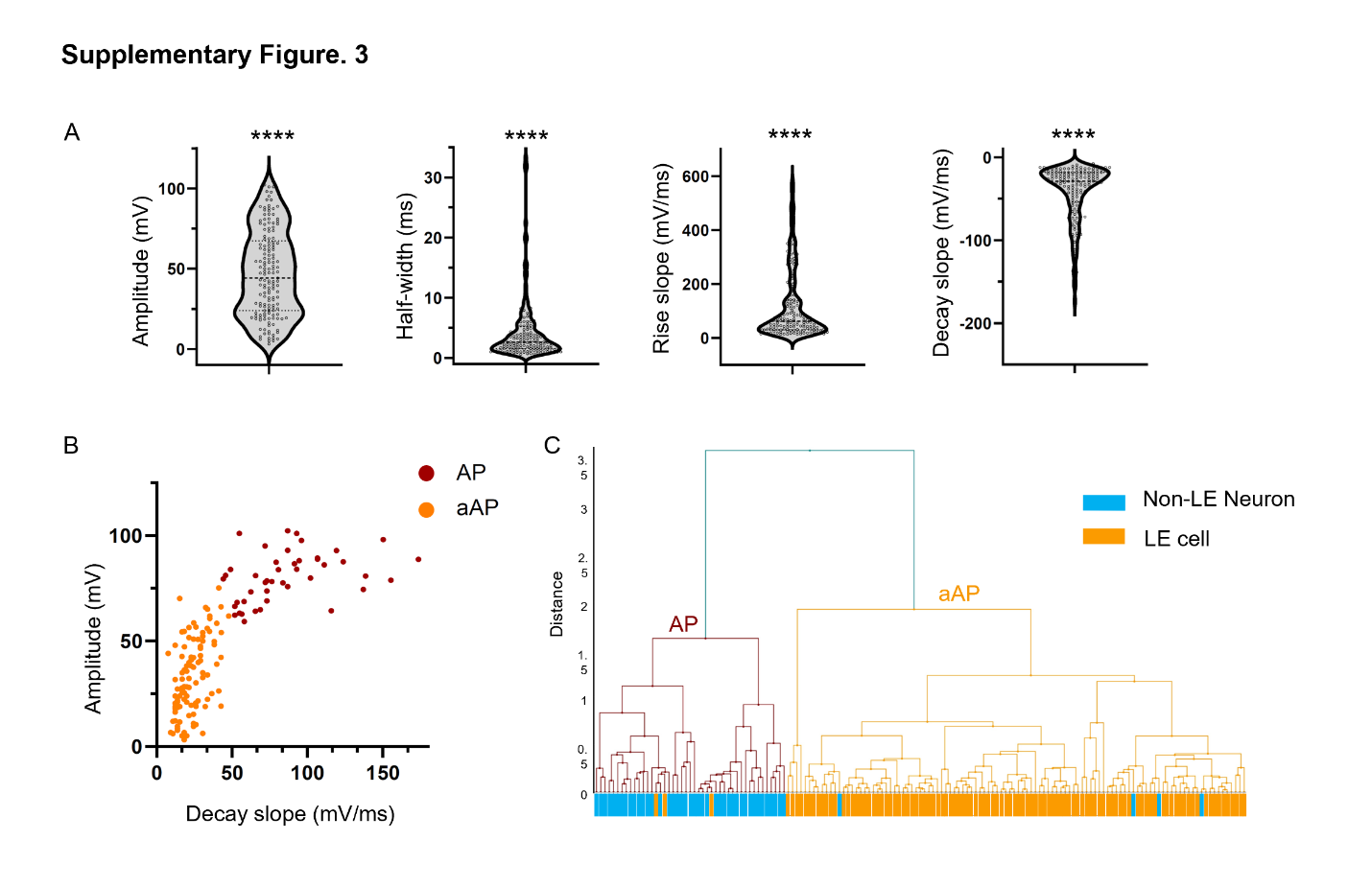
**Supplementary Figure 3. Categorization of AP and aAP. A**, Violin plot of amplitude, half-width, rise slope, and decay slope from all recorded excitable cells. The statistical significance from the D’Agostino–Pearson omnibus normality test demonstrates that the parameters presented are not unimodally distributed. Asterisks indicate significant deviation from a normal distribution (***p < 0.0001). **B**, Scatterplot of amplitude versus decay slope. Cells classified as AP (red) and aAP (orange) phenotypes in the dendrogram are red and yellow, respectively. **C**, Hierarchical cluster analysis was performed on all recorded excitable cells using the electrophysiological parameters shown in B, and the resulting dendrogram displays individual cells along the x-axis and squared Euclidean distances between cells and clusters along the y-axis. This clustering separated APs and aAPs across both non-LE neurons and LE cells, with a small fraction of non-LE neurons exhibiting aAPs (4 cells) and 3 LE cells exhibiting APs.

### Supplementary Figure 4.


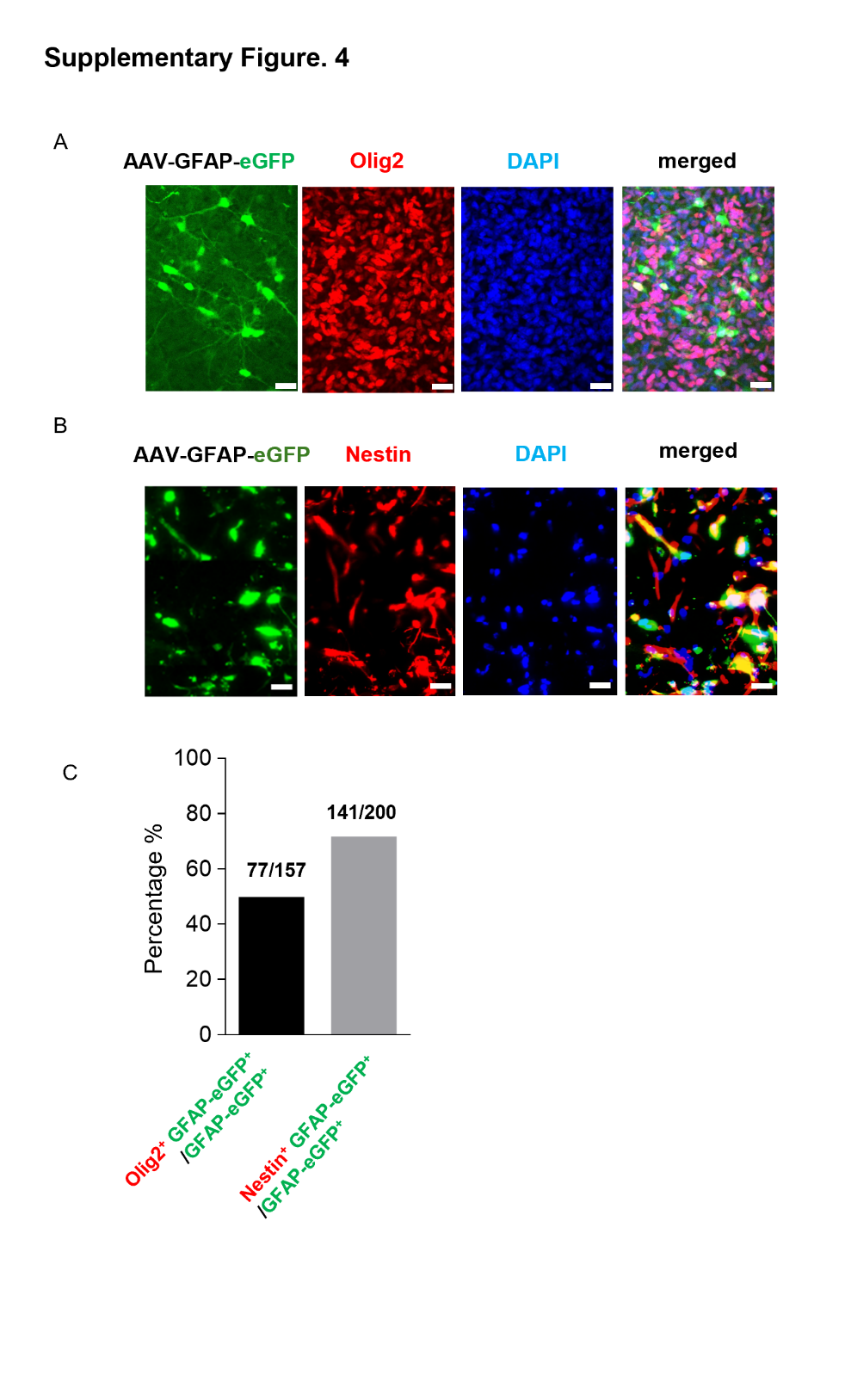


**Supplementary Figure 4.** Specificity of AAV-GFAP–eGFP labeling in tumor-infiltrated tissue. A, Representative images of brain slices transduced with AAV-GFAP–eGFP (green) and immunostained for Olig2 (red) with DAPI (blue). Merged images show partial overlap between GFAP–eGFP+ cells and Olig2+ cells. B, Representative images of AAV-GFAP–eGFP–transduced slices immunostained for Nestin (red) with DAPI (blue). Merged images illustrate co-localization of hGFAP–eGFP+ cells with Nestin. C, Quantification of co-localization. Left, proportion of hGFAP–eGFP+ cells that are Olig2+ (77/157). Right, proportion of hGFAP–eGFP+ cells that are Nestin+ (141/200). Numbers above bars indicate counted cells. Scale bars, 20 µm.

### Supplementary Figure 5.


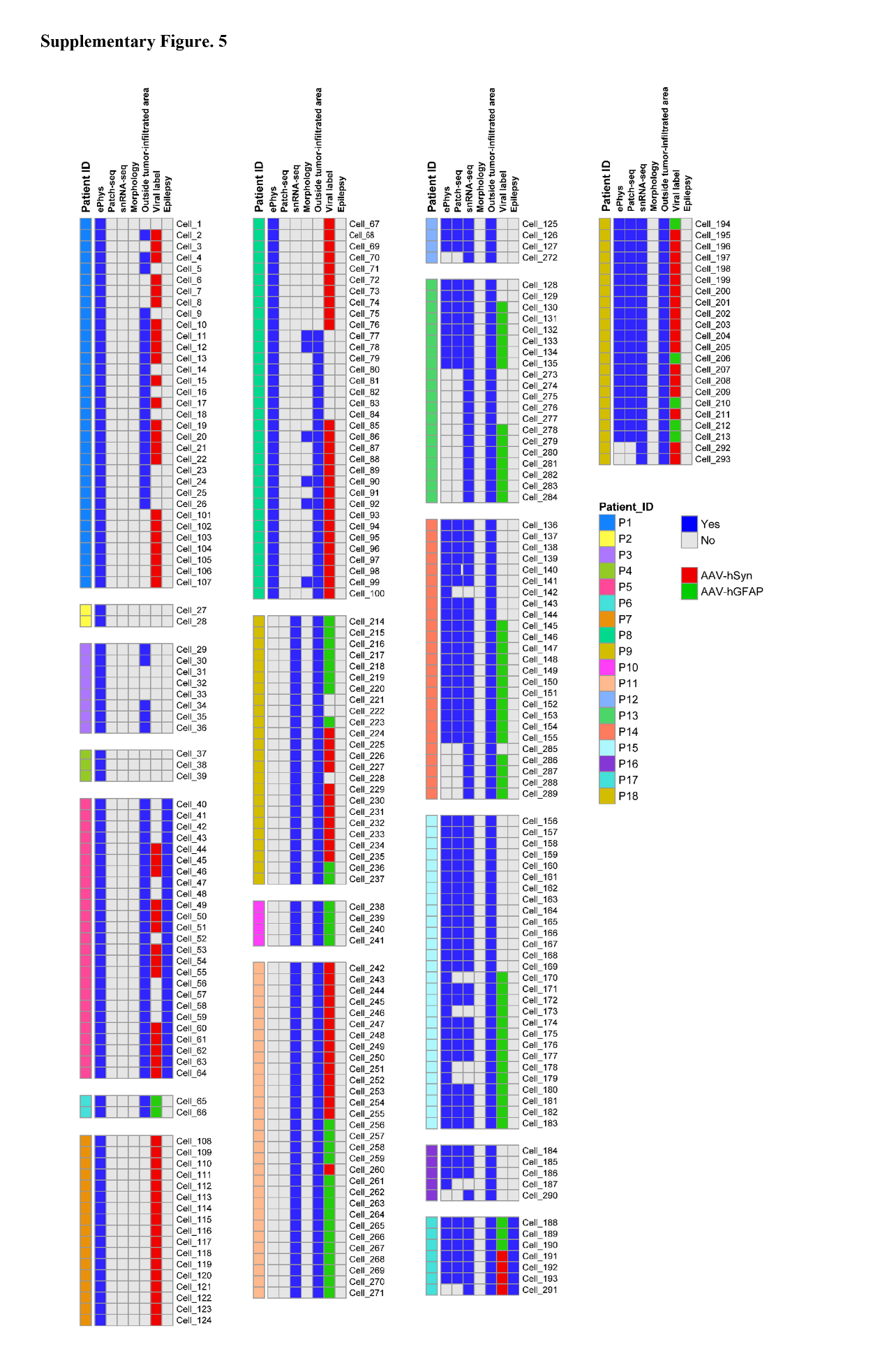


**Supplementary Figure 5. Metadata overview of all recorded cells from glioblastoma patients.** The heatmap summarizes metadata for all 293 individual cells collected from 18 glioblastoma patients in this manuscript. The entire dataset is divided across 18 patients. Each row represents a single cell, annotated by cell ID (left column), and each column indicates experimental metadata, including tissue origin (outside tumor-infiltrated area or within the tumor leading edge), morphology reconstruction (Yes/No), electrophysiological recording (Yes/No), Patch-seq analysis (Yes/No), single-nucleus RNA sequencing (snRNA-seq), and viral labeling (AAV-hSyn-eGFP, marked in red; AAV-hGFAP-eGFP, marked in green). Patient identity is shown as color-coded bars on the far right. The metadata shows which experiments, electrophysiology, transcriptomics, and/or viral labeling, were successfully performed on each individual cell. This integrated overview underscores the heterogeneity of cell sampling and the multimodal nature of single-cell analysis across the patient cohort.

### Supplementary Figure 6.


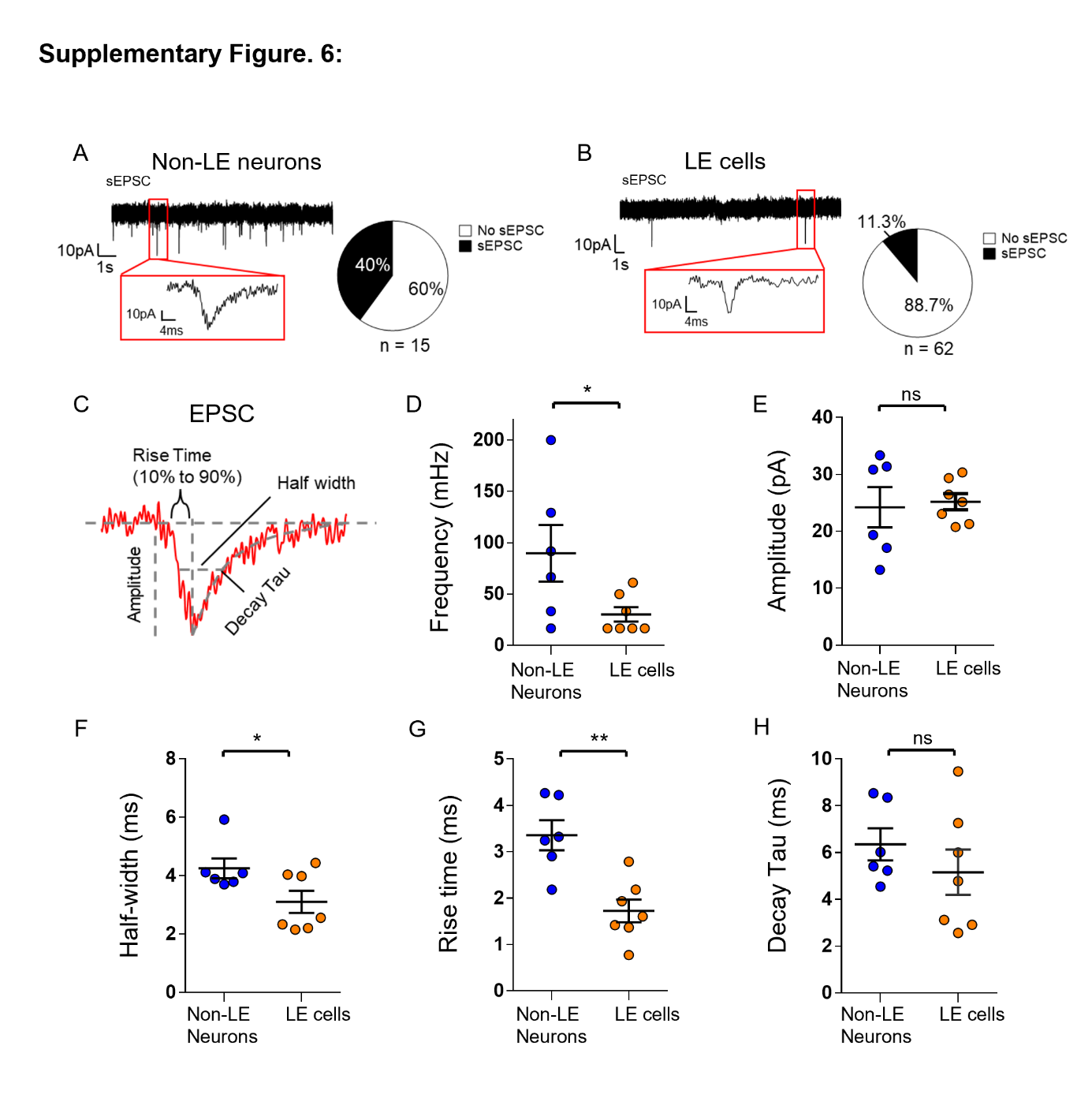


**Supplementary Figure 6. Comparison of excitatory synaptic input between neocortical non-LE neurons and LE cells. A and B,** Representative sEPSC recordings from non-LE Neurons (**A**) and LE cells **(B)**. The red-boxed insets showed a single sEPSC event in the traces. The pie charts showed that **A,** a total of 40% of non-LE neurons had sEPSC detected (6/15 tested neurons: 2/4 neurons from acute and 2/11 neurons from slice cultures). **B**, Among LE cells, 11.3% had detected sEPSC (7/62 tested cells: 1/11 cells from acute and 6/51 cells from slice cultures). **C,** Schematic illustration of a single sEPSC event (red) measured sEPSC properties, including amplitude, half-width, rise time (10%–90%), and decay tau (gray dashed line). **D–H,** Quantitative comparisons between neocortical non-LE neurons (blue) and LE cells (orange) of sEPSC properties. Non-LE neurons (n = 6) exhibited significantly (**D**) higher sEPSC frequency (89.58 ± 27.55 mHz), (**F**) slower half-width (4.25 ± 0.34 ms), (**G**) and longer rise time (3.36 ± 0.33 ms) compared to LE cells (n = 7) (sEPSC frequency: 30.16 ± 7.05 mHz, half-width: 3.10 ± 0.38 ms, rise time: 1.72 ± 0.25 ms). No significant differences were observed in (**E**) sEPSC amplitude (non-LE neurons: 24.22 ± 3.53 pA; LE cells: 25.20 ± 1.42 pA) and (**H**) decay tau (non-LE neurons: 6.35 ± 0.69 ms; LE cells: 5.15 ± 0.97 ms). Statistical significance: *p < 0.05, **p < 0.01.

### Supplementary Figure 7.


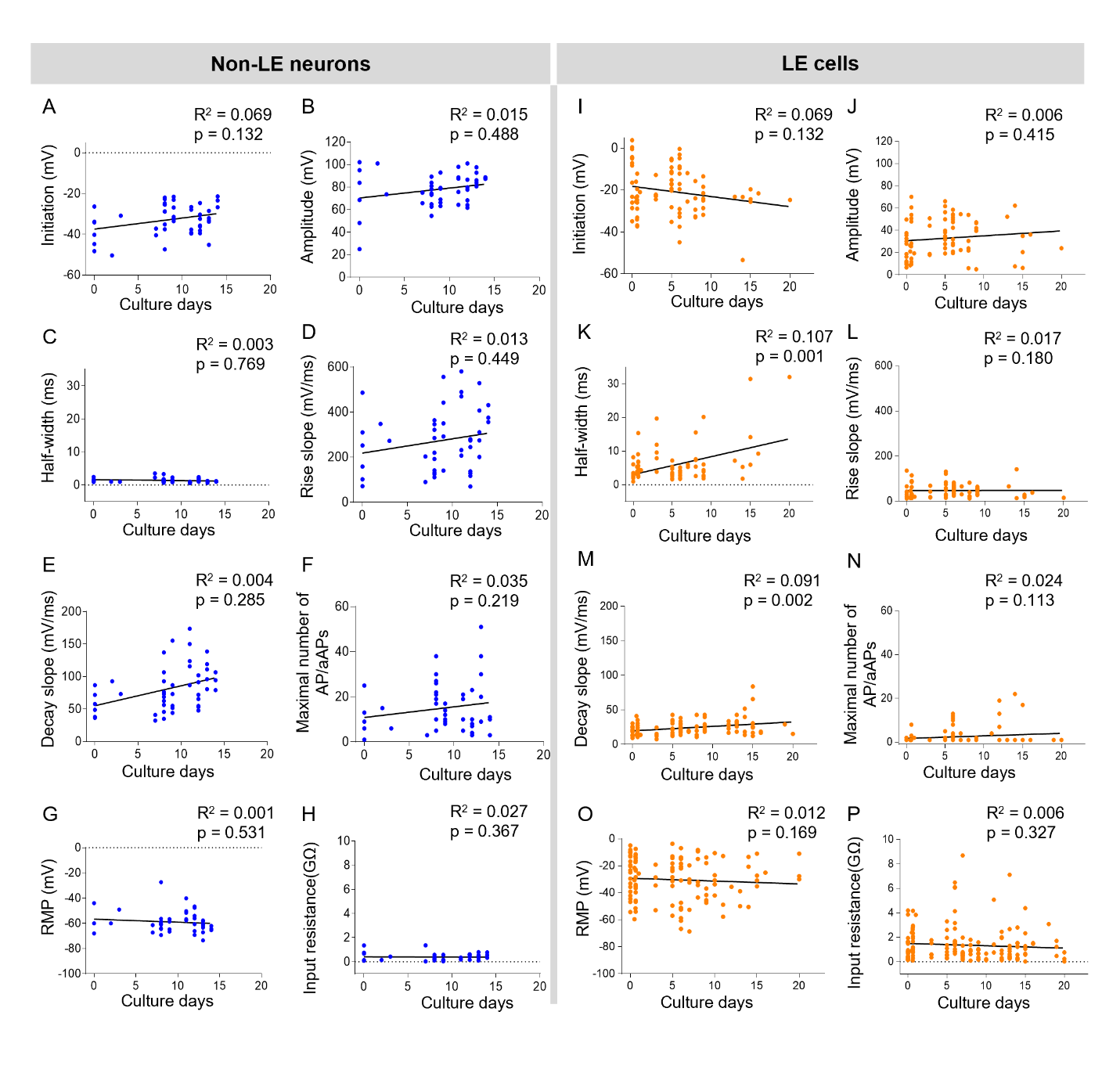
 **Supplementary Figure 7. Dynamic analysis of electrophysiological parameters of AP/aAP over culture time in non-LE neurons and LE cells over the culture period.** The figure displays linear regression analyses of electrophysiological waveform parameters and passive membrane properties over time in culture for neocortical non-LE neurons (left, blue) and LE cells (right, orange). Parameters include initiation, amplitude, half-width, rise slope, decay slope, maximal number of AP/aAPs, resting membrane potential (RMP), and input resistance. Pearson’s correlation and linear regression were used to assess trends over time. **A–H**, Non-LE neurons showed no significant correlation between culture time and any measured parameters (all R² < 0.069, p > 0.05). **I, J, L, M, N, O, P**, LE cells exhibited no consistent significant correlation between culture duration and electrophysiological or passive properties (all R² < 0.065, p > 0.05). **K** and **M**, for LE cells, there are weak correlations in threshold, amplitude, and half-width (R² < 0.107 and p < 0.05) over the culture period. These results suggest that both non-LE neurons and LE cells maintain relatively stable electrophysiological and intrinsic membrane properties during the *ex vivo* culture period.

### Supplementary Figure 8.


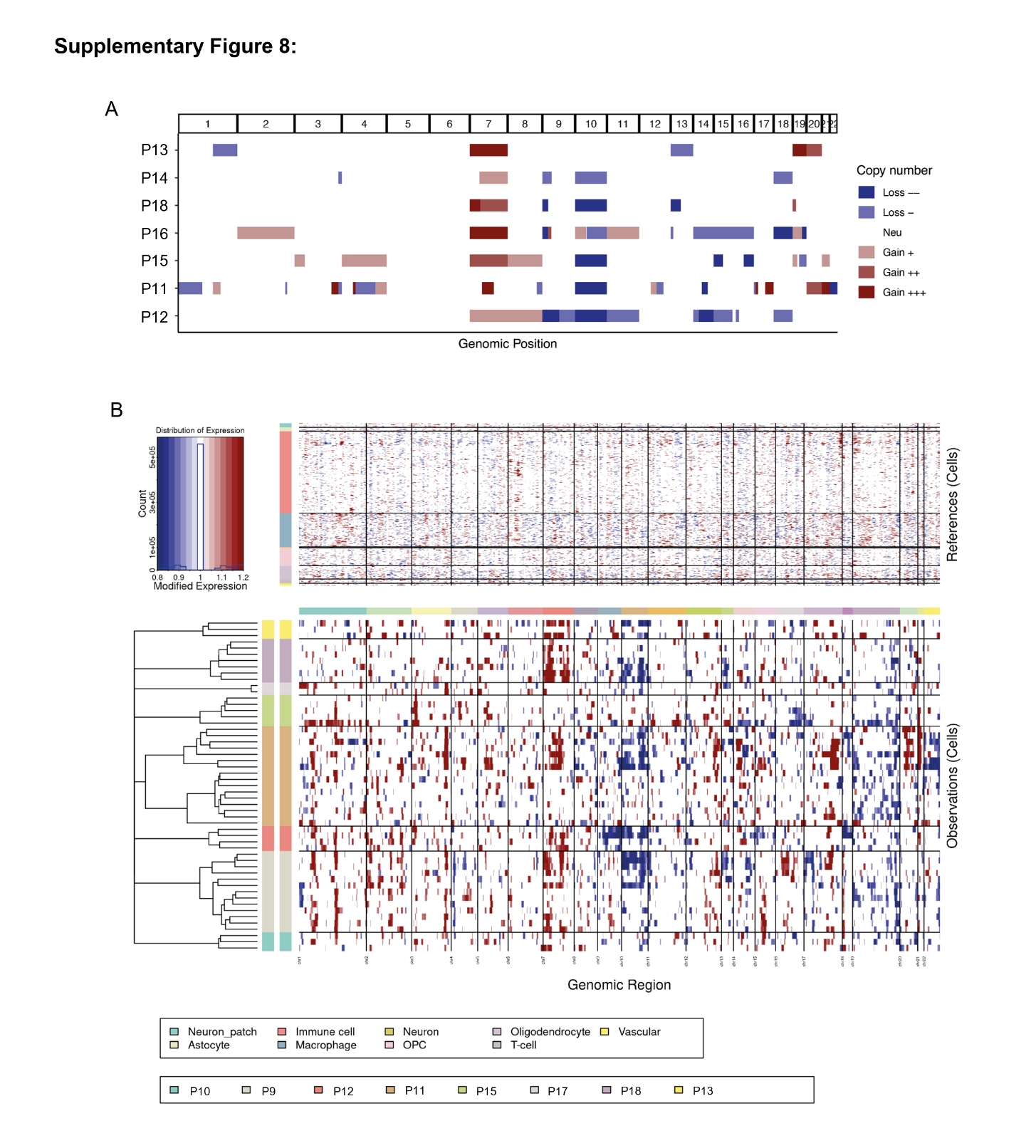


**Supplementary Figure 8.** Copy-number characterization of GBCs. **A**, Genome-wide copy-number profiles of each patient, derived from shallow WGS using ACE[^4^](#_ENREF_4). Colors of the segments denote the copy number status. **B**, Single-cell inferred CNV of GBCs across patients, generated using InferCNV. The upper panel illustrates the normal cells used as reference, including non-tumor cells from our patch-seq LE dataset and additional normal cell types from the Darmanis et al. dataset[^5^](#_ENREF_5). The lower panel displays the GBCs, with the color indicating the inferred copy number level within a cell.

### Supplementary Figure 9.


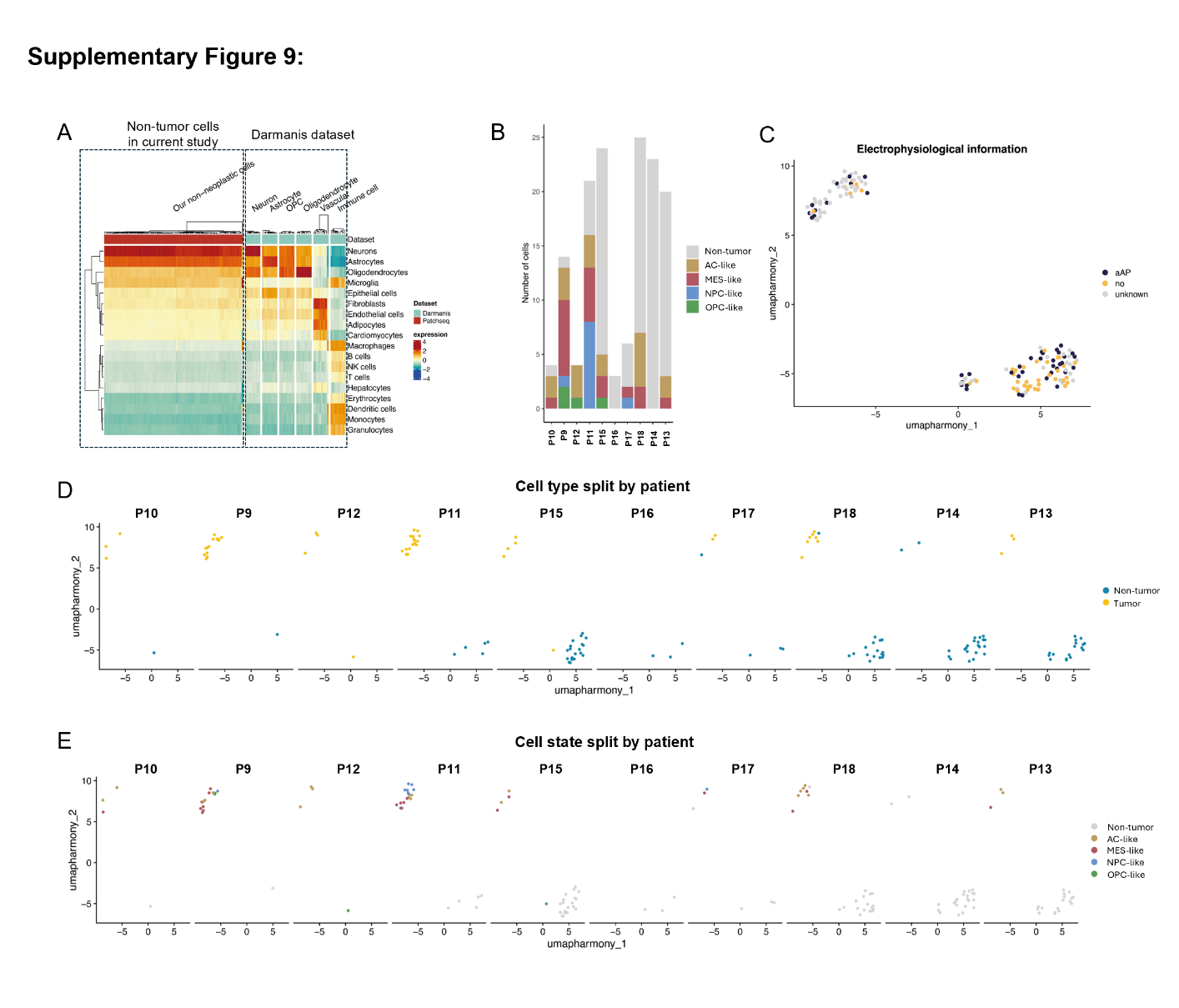


**Supplementary Figure 9. Transcriptomic and electrophysiological characterization of neocortical LE cells. A**, Heatmap showing label transfer of LE cells to reference cell types from the Darmanis dataset, confirming the presence of non-neoplastic neuronal identities, except one microglia cell identified. **B**, A bar plot of inferred cellular states across patients, including AC-like, MES-like, NPC-like, OPC-like, and non-tumor cells. GBCs with different cell states can be sampled within the same patient, and this phenomenon is consistent across patients. **C,** UMAP embedding of LE cells with electrophysiological annotation, showing the distribution of aAP-, no-aAP cells, and nucleus-harvested-only cells with unknown electrophysiological profiles. **D**, UMAP plots showing cell type distribution (tumor vs. non-tumor cells) separated by patient. **E**, UMAP plots showing inferred cellular states of LE cells separated by patient, highlighting heterogeneous tumor cell states (AC-like, MES-like, NPC-like, OPC-like) compared with non-tumor cells.

### Supplementary Figure 10.


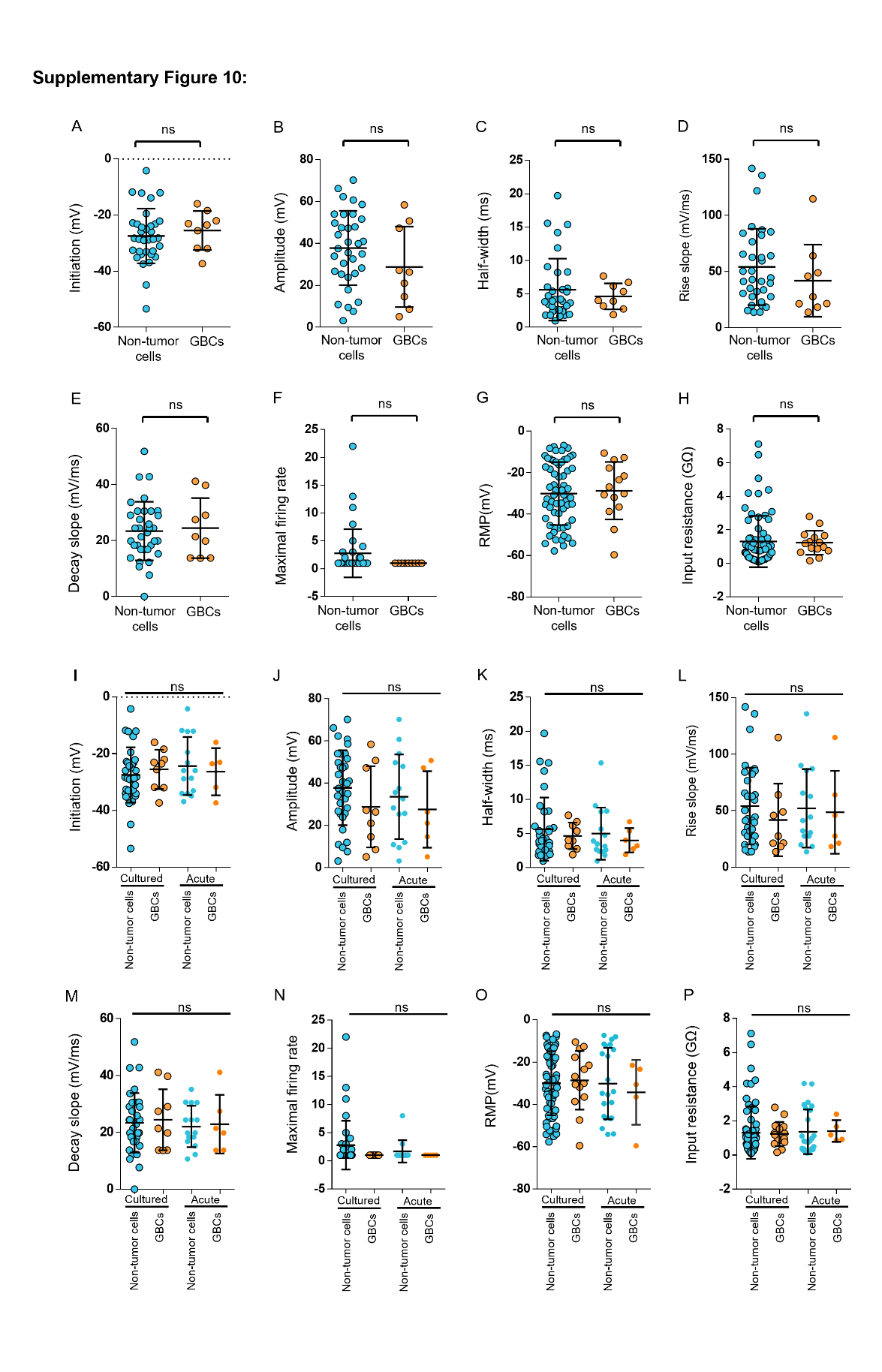


**Supplementary Figure 10. Electrophysiological comparisons between transcriptomically identified non-tumor cells and GBCs. A–H,** Quantitative analysis of electrophysiological properties of Patch-seq recorded cells, classified as non-tumor cells or GBCs based on single-nucleus transcriptomic profiles. Electrophysiological parameters include (**A**) initiation potential, (**B**) amplitude, (**C**) half-width, (**D**) rise slope, (**E**) decay slope, (**F**) maximal number of APs/aAPs, (**G**) RMP, and (**H**) input resistance. No significant differences were observed between non-tumor cells and GBCs for any of the measured parameters. **I–P,** Analysis of non-tumor cells or GBCs recorded from both acute and cultured brain slices. Electrophysiological parameters include (**I**) initiation potential, (**J**) amplitude, (**K**) half-width, (**L**) rise slope, (**M**) decay slope, (**N**) maximal number of APs/aAPs, (**O**) RMP, and (**P)** input resistance. There is no significant difference between non-tumor cells or GBCs in both acute and cultured conditions.

### Supplementary Figure 11.


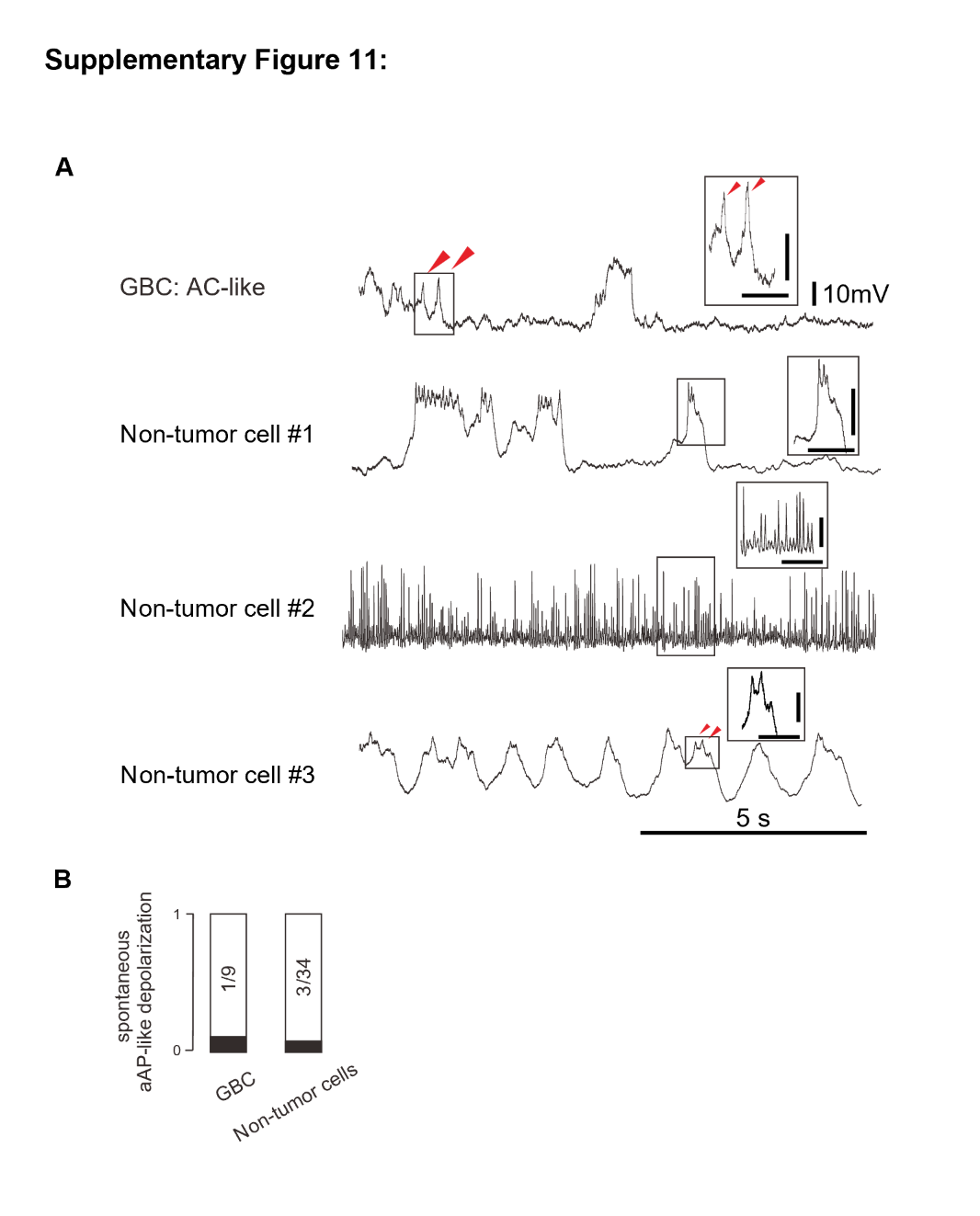


**Supplementary Figure 11. Spontaneous membrane potential activity of LE cells in patch-seq experiments. A**, Resting membrane potential recordings from LE aAP cells acquired in I = 0 current-clamp mode (no current injection). Traces include one GBC with an astrocyte-like (AC-like) state and three non-tumor cells that exhibit spontaneous transient depolarization events. Insets, enlarged views of representative events; Inset scale bars, 10 mV, 1 s. Red arrowheads depict spontaneous depolarization events. **B**, Bar plot of the fraction of aAP-generating LE neurons and LE GBCs that showed spontaneous aAP-like depolarization.

### Supplementary Figure 12.


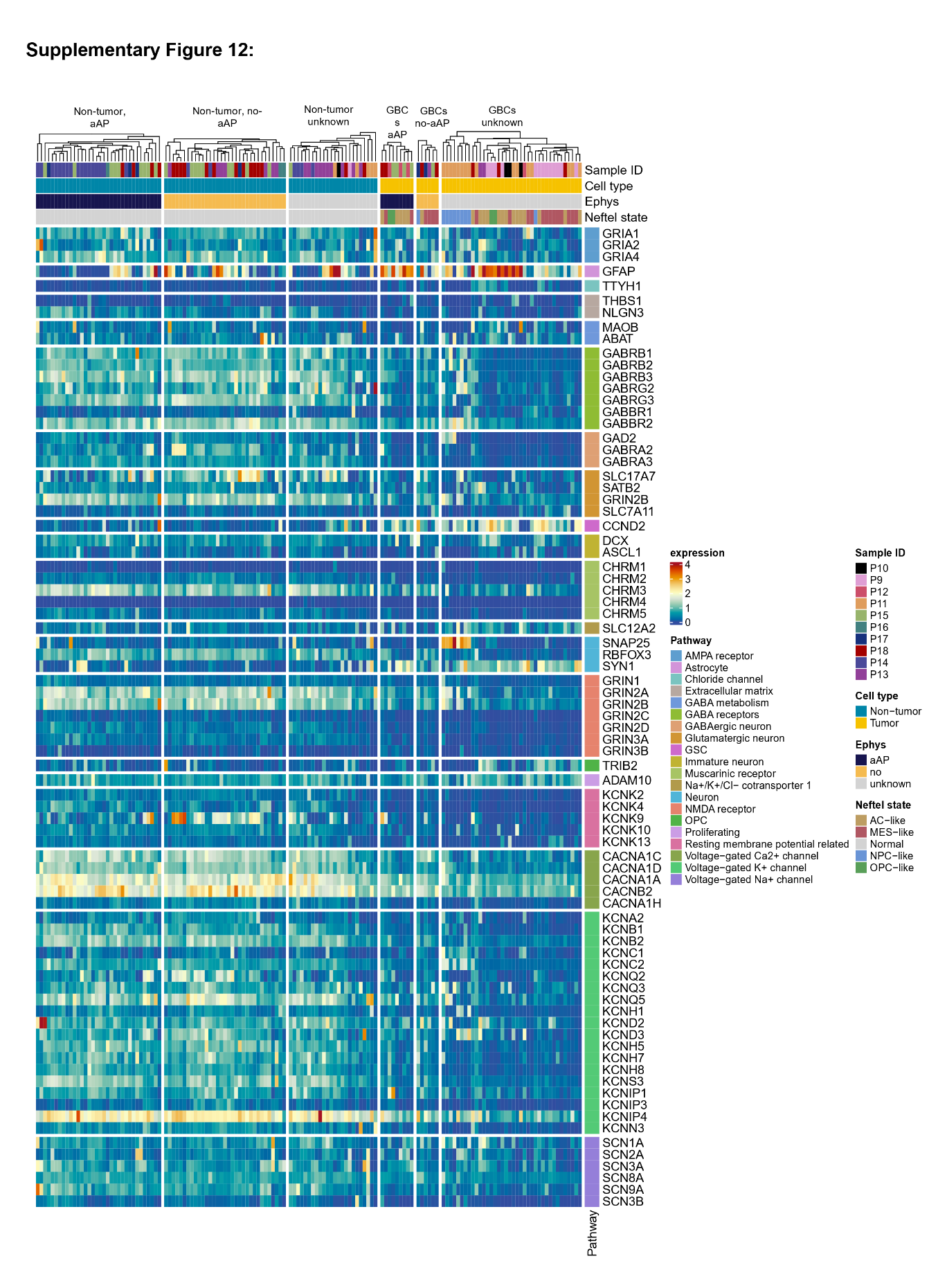


**Supplementary Figure 12. Electrophysiological and functional-related gene expression in non-tumor cells and GBCs.** Heatmap showing the expression of selected genes in Patch-seq samples. Hierarchical clustering separates non-tumor cells and GBCs into distinct groups, including 1. aAP; 2. no-aAP; 3. transcriptomic-only (unknown). Gene families include 1. Neurotransmission: GABA receptors, glutamate receptors, acetylcholine receptors; 2. cell identity: neuron, glutamatergic, GABAergic, immature, astrocyte, OPC, GSC; 3. ion channel: chloride channels and transporters, potassium channels, calcium channels, and sodium channels; 4. Functional signature: extracellular matrix, GABA metabolism, proliferation. Color scale indicates normalized expression levels.

### Supplementary Figure 13.


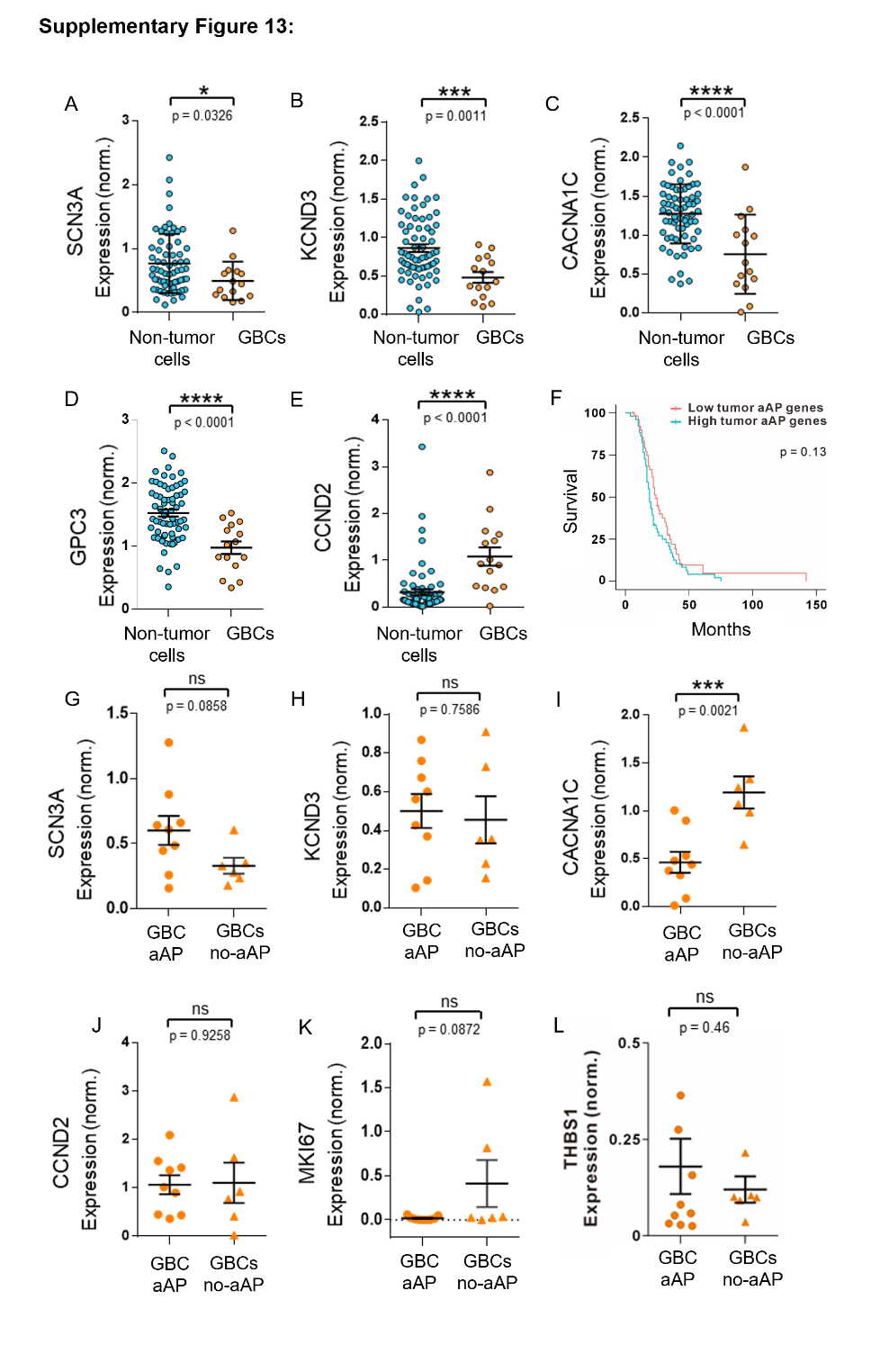


**Supplementary Figure 13. Transcriptomic and physiological correlation of aAP-related channel gene expression in non-tumor cells and GBCs. A**-**E,** Comparison of the expression of selected genes (**A**) SCN3A, (**B**) KCND3, (**C**) CACNA1C, (**D**) GPC3, and (**E**) CCND2 between non-tumor cells (blue circles) and GBCs (orange symbols) with available electrophysiological recordings. **F,** Kaplan–Meier survival curves comparing TCGA patients with high versus low expression of tumor aAP–associated gene signatures, with no significant difference in overall survival. **G–L,** Comparison of the expression of selected genes, (**G**) SCN3A, (**H**) KCND3, (**I**) CACNA1C, (**J**) CCND2, (**K**) MKI67, and (**L**) THBS1, between aAP- (orange circles) and no-aAP GBCs (orange triangle). Statistical significance: *p < 0.05, **p < 0.01, ***p < 0.005, ****p < 0.001.

## Supplementary Tables

### Supplementary Table 1.

| **Cell Number** | **Case ID** | **Slice Type** | **Viral Label** | **Tumor Cell Label** | **Neftel Cell State Label** | **aAP Label** | **UMAP Cluster** |
| --- | --- | --- | --- | --- | --- | --- | --- |
|  |  |  |  |  |  |  |  |
| 1 | P14 | Cultured | AAV-GFAP-eGFP | Non-tumor cell | Normal | aAP | Non-tumor cluster 2 |
| 2 | P14 | Cultured | AAV-GFAP-eGFP | Non-tumor cell | Normal | aAP | Non-tumor cluster 2 |
| 3 | P14 | Cultured | AAV-GFAP-eGFP | Non-tumor cell | Normal | Unknown | Non-tumor cluster 1 |
| 4 | P14 | Cultured | AAV-GFAP-eGFP | Non-tumor cell | Normal | aAP | Non-tumor cluster 1 |
| 5 | P14 | Cultured | AAV-GFAP-eGFP | Non-tumor cell | Normal | Unknown | Non-tumor cluster 1 |
| 6 | P14 | Cultured | AAV-GFAP-eGFP | Non-tumor cell | Normal | aAP | Non-tumor cluster 2 |
| 7 | P14 | Cultured | AAV-GFAP-eGFP | Non-tumor cell | Normal | Unknown | Non-tumor cluster 1 |
| 8 | P14 | Cultured | AAV-GFAP-eGFP | Non-tumor cell | Normal | aAP | Non-tumor cluster 1 |
| 9 | P14 | Cultured | AAV-GFAP-eGFP | Non-tumor cell | Normal | aAP | Non-tumor cluster 1 |
| 10 | P14 | Cultured | AAV-GFAP-eGFP | Non-tumor cell | Normal | no-aAP | Non-tumor cluster 1 |
| 11 | P9 | Cultured | AAV-hSyn1-eGFP | Tumor | NPC-like | Unknown | Tumor cluster 1 |
| 12 | P15 | Acute | None | Non-tumor cell | Normal | no-aAP | Non-tumor cluster 1 |
| 13 | P15 | Acute | None | Non-tumor cell | Normal | aAP | Non-tumor cluster 1 |
| 14 | P15 | Acute | None | Non-tumor cell | Normal | aAP | Non-tumor cluster 1 |
| 15 | P15 | Acute | None | Non-tumor cell | Normal | aAP | Non-tumor cluster 1 |
| 16 | P15 | Acute | None | Tumor | AC-like | aAP | Tumor cluster 2 |
| 17 | P15 | Acute | None | Non-tumor cell | Normal | no-aAP | Non-tumor cluster 1 |
| 18 | P15 | Acute | None | Non-tumor cell | Normal | aAP | Non-tumor cluster 1 |
| 19 | P15 | Acute | None | Non-tumor cell | Normal | aAP | Non-tumor cluster 1 |
| 20 | P15 | Acute | None | Non-tumor cell | Normal | no-aAP | Non-tumor cluster 1 |
| 21 | P9 | Cultured | AAV-hSyn1-eGFP | Tumor | AC-like | Unknown | Tumor cluster 1 |
| 22 | P15 | Acute | None | Tumor | OPC-like | aAP | Tumor cluster 2 |
| 23 | P15 | Acute | None | Non-tumor cell | Normal | no-aAP | Non-tumor cluster 1 |
| 24 | P15 | Acute | None | Non-tumor cell | Normal | no-aAP | Non-tumor cluster 1 |
| 25 | P15 | Acute | None | Non-tumor cell | Normal | no-aAP | Non-tumor cluster 1 |
| 26 | P15 | Acute | None | Non-tumor cell | Normal | no-aAP | Non-tumor cluster 1 |
| 27 | P15 | Cultured | AAV-GFAP-eGFP | Non-tumor cell | Normal | aAP | Non-tumor cluster 1 |
| 28 | P15 | Cultured | AAV-GFAP-eGFP | Non-tumor cell | Normal | Unknown | Non-tumor cluster 1 |
| 29 | P15 | Cultured | AAV-GFAP-eGFP | Non-tumor cell | Normal | aAP | Non-tumor cluster 1 |
| 30 | P9 | Cultured | AAV-hSyn1-eGFP | Tumor | MES-like | Unknown | Tumor cluster 1 |
| 31 | P15 | Cultured | AAV-GFAP-eGFP | Non-tumor cell | Normal | aAP | Non-tumor cluster 1 |
| 32 | P15 | Cultured | AAV-GFAP-eGFP | Tumor | MES-like | aAP | Tumor cluster 1 |
| 33 | P15 | Cultured | AAV-GFAP-eGFP | Non-tumor cell | Normal | no-aAP | Non-tumor cluster 1 |
| 34 | P15 | Cultured | AAV-GFAP-eGFP | Tumor | AC-like | aAP | Tumor cluster 1 |
| 35 | P15 | Cultured | AAV-GFAP-eGFP | Non-tumor cell | Normal | aAP | Non-tumor cluster 1 |
| 36 | P15 | Cultured | AAV-GFAP-eGFP | Tumor | MES-like | no-aAP | Tumor cluster 2 |
| 37 | P15 | Cultured | AAV-GFAP-eGFP | Non-tumor cell | Normal | no-aAP | Non-tumor cluster 1 |
| 38 | P9 | Cultured | AAV-hSyn1-eGFP | Tumor | MES-like | Unknown | Tumor cluster 1 |
| 39 | P16 | Acute | None | Non-tumor cell | Normal | no-aAP | Non-tumor cluster 2 |
| 40 | P16 | Acute | None | Non-tumor cell | Normal | no-aAP | Non-tumor cluster 1 |
| 41 | P16 | Acute | None | Non-tumor cell | Normal | aAP | Non-tumor cluster 1 |
| 42 | P17 | Cultured | AAV-GFAP-eGFP | Tumor | NPC-like | no-aAP | Tumor cluster 2 |
| 43 | P17 | Cultured | AAV-GFAP-eGFP | Non-tumor cell | Normal | aAP | Non-tumor cluster 2 |
| 44 | P17 | Cultured | AAV-GFAP-eGFP | Non-tumor cell | Normal | aAP | Non-tumor cluster 1 |
| 45 | P17 | Cultured | AAV-hSyn1-eGFP | Non-tumor cell | Normal | aAP | Non-tumor cluster 1 |
| 46 | P17 | Cultured | AAV-hSyn1-eGFP | Non-tumor cell | Normal | no-aAP | Non-tumor cluster 1 |
| 47 | P17 | Cultured | AAV-hSyn1-eGFP | Tumor | MES-like | no-aAP | Tumor cluster 2 |
| 48 | P18 | Cultured | AAV-GFAP-eGFP | Non-tumor cell | Normal | no-aAP | Non-tumor cluster 1 |
| 49 | P18 | Cultured | AAV-hSyn1-eGFP | Tumor | AC-like | no-aAP | Tumor cluster 2 |
| 50 | P18 | Cultured | AAV-hSyn1-eGFP | Non-tumor cell | Normal | aAP | Non-tumor cluster 1 |
| 51 | P18 | Cultured | AAV-hSyn1-eGFP | Tumor | AC-like | Unknown | Tumor cluster 2 |
| 52 | P18 | Cultured | AAV-hSyn1-eGFP | Non-tumor cell | Normal | no-aAP | Non-tumor cluster 1 |
| 53 | P18 | Cultured | AAV-hSyn1-eGFP | Non-tumor cell | Normal | no-aAP | Non-tumor cluster 1 |
| 54 | P18 | Cultured | AAV-hSyn1-eGFP | Non-tumor cell | Normal | no-aAP | Non-tumor cluster 1 |
| 55 | P18 | Cultured | AAV-hSyn1-eGFP | Non-tumor cell | Normal | Unknown | Non-tumor cluster 2 |
| 56 | P9 | Cultured | AAV-hSyn1-eGFP | Tumor | MES-like | Unknown | Tumor cluster 1 |
| 57 | P18 | Cultured | AAV-hSyn1-eGFP | Non-tumor cell | Normal | aAP | Non-tumor cluster 2 |
| 58 | P18 | Cultured | AAV-hSyn1-eGFP | Non-tumor cell | Normal | no-aAP | Non-tumor cluster 1 |
| 59 | P18 | Cultured | AAV-hSyn1-eGFP | Non-tumor cell | Normal | no-aAP | Non-tumor cluster 1 |
| 60 | P18 | Cultured | AAV-hSyn1-eGFP | Non-tumor cell | Normal | Unknown | Non-tumor cluster 1 |
| 61 | P18 | Cultured | AAV-hSyn1-eGFP | Tumor | MES-like | aAP | Tumor cluster 1 |
| 62 | P18 | Cultured | AAV-hSyn1-eGFP | Non-tumor cell | Normal | no-aAP | Non-tumor cluster 1 |
| 63 | P18 | Cultured | AAV-GFAP-eGFP | Tumor | AC-like | Unknown | Tumor cluster 1 |
| 64 | P18 | Cultured | AAV-hSyn1-eGFP | Non-tumor cell | Normal | no-aAP | Non-tumor cluster 1 |
| 65 | P18 | Cultured | AAV-GFAP-eGFP | Non-tumor cell | Normal | no-aAP | Non-tumor cluster 1 |
| 66 | P18 | Cultured | AAV-hSyn1-eGFP | Non-tumor cell | Normal | aAP | Non-tumor cluster 1 |
| 67 | P9 | Cultured | AAV-hSyn1-eGFP | Tumor | OPC-like | Unknown | Tumor cluster 2 |
| 68 | P18 | Cultured | AAV-hSyn1-eGFP | Non-tumor cell | Normal | no-aAP | Non-tumor cluster 1 |
| 69 | P18 | Cultured | AAV-hSyn1-eGFP | Tumor | AC-like | aAP | Tumor cluster 1 |
| 70 | P18 | Cultured | AAV-GFAP-eGFP | Non-tumor cell | Normal | no-aAP | Non-tumor cluster 1 |
| 71 | P18 | Cultured | AAV-hSyn1-eGFP | Tumor | MES-like | no-aAP | Tumor cluster 2 |
| 72 | P18 | Cultured | AAV-hSyn1-eGFP | Tumor | AC-like | Unknown | Tumor cluster 2 |
| 73 | P18 | Cultured | AAV-GFAP-eGFP | Non-tumor cell | Normal | no-aAP | Non-tumor cluster 1 |
| 74 | P18 | Cultured | AAV-GFAP-eGFP | Non-tumor cell | Normal | aAP | Non-tumor cluster 1 |
| 75 | P9 | Cultured | AAV-hSyn1-eGFP | Tumor | OPC-like | Unknown | Tumor cluster 2 |
| 76 | P9 | Cultured | AAV-hSyn1-eGFP | Tumor | AC-like | Unknown | Tumor cluster 2 |
| 77 | P9 | Cultured | AAV-GFAP-eGFP | Tumor | AC-like | Unknown | Tumor cluster 1 |
| 78 | P9 | Cultured | AAV-hSyn1-eGFP | Tumor | MES-like | Unknown | Tumor cluster 1 |
| 79 | P9 | Cultured | AAV-hSyn1-eGFP | Tumor | MES-like | Unknown | Tumor cluster 2 |
| 80 | P10 | Cultured | AAV-GFAP-eGFP | Non-tumor cell | Normal | Unknown | Non-tumor cluster 2 |
| 81 | P10 | Cultured | AAV-GFAP-eGFP | Tumor | AC-like | Unknown | Tumor cluster 1 |
| 82 | P10 | Cultured | AAV-GFAP-eGFP | Tumor | MES-like | Unknown | Tumor cluster 1 |
| 83 | P10 | Cultured | AAV-GFAP-eGFP | Tumor | AC-like | Unknown | Tumor cluster 2 |
| 84 | P11 | Cultured | AAV-hSyn1-eGFP | Non-tumor cell | Normal | Unknown | Non-tumor cluster 1 |
| 85 | P11 | Cultured | AAV-hSyn1-eGFP | Tumor | NPC-like | Unknown | Tumor cluster 2 |
| 86 | P11 | Cultured | AAV-hSyn1-eGFP | Tumor | NPC-like | Unknown | Tumor cluster 2 |
| 87 | P11 | Cultured | AAV-hSyn1-eGFP | Tumor | NPC-like | Unknown | Tumor cluster 2 |
| 88 | P11 | Cultured | AAV-hSyn1-eGFP | Non-tumor cell | Normal | Unknown | Non-tumor cluster 2 |
| 89 | P11 | Cultured | AAV-hSyn1-eGFP | Tumor | NPC-like | Unknown | Tumor cluster 2 |
| 90 | P11 | Cultured | AAV-hSyn1-eGFP | Tumor | NPC-like | Unknown | Tumor cluster 2 |
| 91 | P11 | Cultured | AAV-hSyn1-eGFP | Non-tumor cell | Normal | Unknown | Non-tumor cluster 2 |
| 92 | P11 | Cultured | AAV-hSyn1-eGFP | Tumor | NPC-like | Unknown | Tumor cluster 1 |
| 93 | P11 | Cultured | AAV-hSyn1-eGFP | Tumor | NPC-like | Unknown | Tumor cluster 2 |
| 94 | P11 | Cultured | AAV-hSyn1-eGFP | Non-tumor cell | Normal | Unknown | Non-tumor cluster 1 |
| 95 | P11 | Cultured | AAV-GFAP-eGFP | Tumor | AC-like | Unknown | Tumor cluster 2 |
| 96 | P11 | Cultured | AAV-hSyn1-eGFP | Tumor | NPC-like | Unknown | Tumor cluster 1 |
| 97 | P9 | Cultured | AAV-GFAP-eGFP | Non-tumor cell | Normal | Unknown | Non-tumor cluster 1 |
| 98 | P11 | Cultured | AAV-GFAP-eGFP | Tumor | AC-like | Unknown | Tumor cluster 1 |
| 99 | P11 | Cultured | AAV-GFAP-eGFP | Non-tumor cell | Normal | Unknown | Non-tumor cluster 1 |
| 100 | P11 | Cultured | AAV-GFAP-eGFP | Tumor | MES-like | Unknown | Tumor cluster 1 |
| 101 | P11 | Cultured | AAV-GFAP-eGFP | Tumor | AC-like | Unknown | Tumor cluster 2 |
| 102 | P11 | Cultured | AAV-GFAP-eGFP | Tumor | MES-like | Unknown | Tumor cluster 2 |
| 103 | P11 | Cultured | AAV-GFAP-eGFP | Tumor | MES-like | Unknown | Tumor cluster 1 |
| 104 | P11 | Cultured | AAV-GFAP-eGFP | Tumor | MES-like | Unknown | Tumor cluster 1 |
| 105 | P11 | Cultured | AAV-GFAP-eGFP | Tumor | MES-like | Unknown | Tumor cluster 1 |
| 106 | P12 | Acute | None | Tumor | OPC-like | aAP | Tumor cluster 2 |
| 107 | P9 | Cultured | AAV-GFAP-eGFP | Tumor | MES-like | Unknown | Tumor cluster 1 |
| 108 | P12 | Acute | None | Tumor | AC-like | aAP | Tumor cluster 2 |
| 109 | P12 | Acute | None | Tumor | AC-like | Unknown | Tumor cluster 2 |
| 110 | P12 | Acute | None | Tumor | AC-like | aAP | Tumor cluster 1 |
| 111 | P13 | Acute | None | Non-tumor cell | Normal | Unknown | Non-tumor cluster 1 |
| 112 | P13 | Acute | None | Non-tumor cell | Normal | no-aAP | Non-tumor cluster 1 |
| 113 | P13 | Acute | None | Non-tumor cell | Normal | Unknown | Non-tumor cluster 1 |
| 114 | P13 | Acute | None | Non-tumor cell | Normal | no-aAP | Non-tumor cluster 1 |
| 115 | P13 | Cultured | None | Non-tumor cell | Normal | Unknown | Non-tumor cluster 2 |
| 116 | P13 | Cultured | None | Non-tumor cell | Normal | Unknown | Non-tumor cluster 1 |
| 117 | P13 | Cultured | None | Non-tumor cell | Normal | Unknown | Non-tumor cluster 2 |
| 118 | P9 | Cultured | AAV-GFAP-eGFP | Tumor | MES-like | Unknown | Tumor cluster 1 |
| 119 | P13 | Cultured | AAV-GFAP-eGFP | Non-tumor cell | Normal | Unknown | Non-tumor cluster 1 |
| 120 | P13 | Cultured | AAV-GFAP-eGFP | Non-tumor cell | Normal | Unknown | Non-tumor cluster 1 |
| 121 | P13 | Cultured | AAV-GFAP-eGFP | Non-tumor cell | Normal | no-aAP | Non-tumor cluster 1 |
| 122 | P13 | Cultured | AAV-GFAP-eGFP | Non-tumor cell | Normal | Unknown | Non-tumor cluster 1 |
| 123 | P13 | Cultured | AAV-GFAP-eGFP | Non-tumor cell | Normal | Unknown | Non-tumor cluster 2 |
| 124 | P13 | Cultured | AAV-GFAP-eGFP | Tumor | MES-like | no-aAP | Tumor cluster 1 |
| 125 | P13 | Cultured | AAV-GFAP-eGFP | Non-tumor cell | Normal | no-aAP | Non-tumor cluster 2 |
| 126 | P13 | Cultured | AAV-GFAP-eGFP | Tumor | AC-like | Unknown | Tumor cluster 2 |
| 127 | P13 | Cultured | AAV-GFAP-eGFP | Tumor | AC-like | Unknown | Tumor cluster 2 |
| 128 | P13 | Cultured | AAV-GFAP-eGFP | Non-tumor cell | Normal | Unknown | Non-tumor cluster 1 |
| 129 | P13 | Cultured | AAV-GFAP-eGFP | Non-tumor cell | Normal | no-aAP | Non-tumor cluster 1 |
| 130 | P13 | Cultured | AAV-GFAP-eGFP | Non-tumor cell | Normal | no-aAP | Non-tumor cluster 1 |
| 131 | P13 | Cultured | AAV-GFAP-eGFP | Non-tumor cell | Normal | no-aAP | Non-tumor cluster 1 |
| 132 | P14 | Acute | None | Non-tumor cell | Normal | no-aAP | Non-tumor cluster 1 |
| 133 | P14 | Acute | None | Non-tumor cell | Normal | aAP | Non-tumor cluster 1 |
| 134 | P14 | Acute | None | Non-tumor cell | Normal | aAP | Non-tumor cluster 1 |
| 135 | P14 | Acute | None | Non-tumor cell | Normal | aAP | Non-tumor cluster 1 |
| 136 | P14 | Acute | None | Non-tumor cell | Normal | Unknown | Non-tumor cluster 1 |
| 137 | P14 | Acute | None | Non-tumor cell | Normal | aAP | Non-tumor cluster 1 |
| 138 | P14 | Acute | None | Non-tumor cell | Normal | aAP | Non-tumor cluster 1 |
| 139 | P14 | Acute | None | Non-tumor cell | Normal | aAP | Non-tumor cluster 1 |
| 140 | P14 | Acute | None | Non-tumor cell | Normal | aAP | Non-tumor cluster 1 |
| 141 | P14 | Cultured | AAV-GFAP-eGFP | Non-tumor cell | Normal | aAP | Non-tumor cluster 2 |
| 142 | P14 | Cultured | AAV-GFAP-eGFP | Non-tumor cell | Normal | aAP | Non-tumor cluster 1 |
| 143 | P14 | Cultured | AAV-GFAP-eGFP | Non-tumor cell | Normal | aAP | Non-tumor cluster 1 |
| 144 | P14 | Cultured | AAV-GFAP-eGFP | Non-tumor cell | Normal | aAP | Non-tumor cluster 1 |
|  |  |  |  |  |  |  |  |

**Supplementary Table 1. A list of integrated annotations of individual LE cells from Patch-seq and nuclei harvesting experiments.** This table presents individual cell annotations derived from both Patch-seq and single-nucleus harvesting experiments performed on cells within the glioblastoma tumor leading edge. Each row represents a single cell, annotated with the corresponding patient case, gene signature enrichment (e.g., tumor cell, non-tumor cell), aAP phenotype, and additional classifications used for UMAP visualization. The integrated transcriptomic and electrophysiological profiles were used to distinguish glioblastoma cells (GBCs) and non-neoplastic cells. These annotations formed the basis for UMAP projections and group-wise comparisons.

### Supplementary Table 2.

|  | **aAP (n=9)** | **no-aAP (n=6)** | **Unknown (n=38)** |
| --- | --- | --- | --- |
| **AC-like** | 5 | 1 | 14 |
| **MES-like** | 2 | 4 | 13 |
| **NPC-like** | 0 | 1 | 9 |
| **OPC-like** | 2 | 0 | 2 |

**Supplementary Table 2. Summary of the cell state proportion of patch-seq identified LE tumor cells.**

### Supplementary Table 3.

|  | **Curry et al., 2024** | **Present Study** |
| --- | --- | --- |
| **Glioma Type** | IDH-mutant (7 patients), IDH-wt (2 patients), non-tumor sample (1 patient) | IDH-wt (18 patients) |
| **Slice Configuration** | Acute brain slice | Acute brain slice;  Organotypic slice culture |
| **Soma Location of Patch-seq Sampled Cells** | Not described | Tumor-infiltrated neocortex  Tumor leading-edge |
| **Tumor vs. Non-tumor Determination Strategy** | SCRAM | InferCNV validated against matched shallow WGS and Cancer Cell Atlas (Darmanis et al, 2017) |
| **Morphology Features** | Hybrid Glial/NPC morphology | Hybrid Glial/NPC-like morphology |
| **Molecular Features**  **of aAP Generating Cells** | GABA-OPC tumor and non-tumor cells | Tumor cells: AC-like; OPC-like; NPC-like; MES-like;  Non-tumor hybrid cells |
| **Passive electrical features**  **upon current injection**  Resting Membrane Potential  Input Resistance | n.a.  Spiking hybrid cells show higher input resistances than neurons or non-spiking cells | LE cells are more depolarized than non-LE neurons. -27.84 ± 2.23 mV.  LE cells are higher than non-LE neurons. 1.41 ± 0.17 GΩ. |
| **Active electrical properties**  **upon current injection**    AP threshold  AP amplitude  AP half width  AP maximum rise slope  AP maximum decay slope  Maximum number of APs | Spiking behavior observed in IDH-mut cells but not IDH-wt cells  n.a.  Not significantly different from non-tumor cells.  n.a.  n.a.  Mostly single AP, except multiple APs from a Olig2+ pyramidal cell-like GABA-OPC | Similar spiking behavior was observed in IDH-wt tumor cells and non-tumor cells  -19.65 ± 2.35 mV  26.97 ± 3.38 mV  4.40 ± 0.55 ms  42.63 ± 6.17 mV/ms  20.23 ± 1.55 mV/ms  1.39 ± 0.26 APs  Single AP in GBCs and most non-tumor cells. |
| **Pharmacological validation** | TTX confirmed voltage-gated sodium channel mechanism in aAPs | TTX confirmed voltage-gated sodium channel mechanism in aAPs.  TEA confirmed voltage-gated potassium channel mechanism in aAPs. |
| **Spontaneous vs. evoked events** | Evoked single small APs in most cases, except for multiple APs from an Olig2+ pyramidal cell-like GABA-OPC.  Spontaneous activity is not assessed. | Evoked single aAPs in most cases Spontaneous aAP-like transient depolarization recorded  Spontaneous EPSC recorded |

**Supplementary Table 3. Comparison of study design, cell identity criteria, and electrophysiological characterization between Curry et al. (2024) and the present study.** The table summarizes key similarities and differences in glioma type, slice preparation, anatomical sampling location for Patch-seq, tumor versus non-tumor classification strategy, morphological and molecular features of aAP cells, and passive and active membrane properties measured during current injection. Values are reported as mean ± SEM where indicated, and “n.a.” denotes not assessed or not reported. (aAP: aberrant action potential; inferCNV: Inference of copy number variation from single cell RNA seq data; WGS: Whole genome sequencing; TTX: Tetrodotoxin; TEA: Tetraethylammonium; EPSC: Excitatory postsynaptic current; NPC: Neural progenitor cell; OPC: Oligodendrocyte progenitor cell).
